# Improving economic access to healthy diets in first nations communities in high-income, colonised countries: a systematic scoping review

**DOI:** 10.1186/s12937-023-00895-0

**Published:** 2024-01-16

**Authors:** Amanda J. Lee, Lisa-Maree Herron, Stephan Rainow, Lisa Wells, Ingrid Kenny, Leon Kenny, Imogen Wells, Margaret Kavanagh, Suzanne Bryce, Liza Balmer

**Affiliations:** 1 School of Public Health, The University of Queensland, 288 Herston Rd, Herston, QLD 4029 Australia; 2 Nganampa Health Council, 3 Wilkinson St, Alice Springs, NT 0871 Australia; 3 Anangu research team, Ngaanyatjarra Pitjantjatjara Yankunytjatjara (NPY) Women’s Council, 3 Wilkinson St, Alice Springs, NT 0871 Australia; 4 Ngaanyatjarra Pitjantjatjara Yankunytjatjara (NPY) Women’s Council, 3 Wilkinson St, Alice Springs, NT 0871 Australia

**Keywords:** First nations communities, Food security, Economic access, Affordability, Policy, Intervention, Systematic scoping review

## Abstract

**Background:**

Affordability of healthy food is a key determinant of the diet-related health of First Nations Peoples. This systematic scoping review was commissioned by the Ngaanyatjarra Pitjantjatjara Yankunytjatjara Women’s Council (NPYWC) in Central Australia to identify interventions to improve economic access to healthy food in First Nations communities in selected high-income, colonised countries.

**Methods:**

Eight databases and 22 websites were searched to identify studies of interventions and policies to improve economic access to healthy food in First Nations communities in Australia, Canada, the United States or New Zealand from 1996 to May 2022. Data from full text of articles meeting inclusion criteria were extracted to a spreadsheet. Results were collated by descriptive synthesis. Findings were examined with members of the NPYWC Anangu research team at a co-design workshop.

**Results:**

Thirty-five publications met criteria for inclusion, mostly set in Australia (37%) or the US (31%). Interventions (*n* = 21) were broadly categorised as price discounts on healthy food sold in communities (*n* = 7); direct subsidies to retail stores, suppliers and producers (*n* = 2); free healthy food and/or food vouchers provided to community members (*n* = 7); increased financial support to community members (*n* = 1); and other government strategies (*n* = 4).

Promising initiatives were: providing a box of food and vouchers for fresh produce; prescriptions for fresh produce; provision/promotion of subsidised healthy meals and snacks in community stores; direct funds transfer for food for children; offering discounted healthy foods from a mobile van; and programs increasing access to traditional foods. Providing subsidies directly to retail stores, suppliers and producers was least effective.

Identified enablers of effective programs included community co-design and empowerment; optimal promotion of the program; and targeting a wide range of healthy foods, particularly traditional foods where possible. Common barriers in the least successful programs included inadequate study duration; inadequate subsidies; lack of supporting resources and infrastructure for cooking, food preparation and storage; and imposition of the program on communities.

**Conclusions:**

The review identified 21 initiatives aimed at increasing affordability of healthy foods in First Nations communities, of which six were deemed promising. Five reflected the voices and experiences of members of the NPYWC Anangu research team and will be considered by communities for trial in Central Australia. Findings also highlight potential approaches to improve economic access to healthy foods in First Nations communities in other high-income colonised countries.

**Trial registration:**

PROSPERO CRD42022328326.

**Supplementary Information:**

The online version contains supplementary material available at 10.1186/s12937-023-00895-0.

## Background

Aboriginal and Torres Strait Islander peoples (Australia’s First Nations Peoples) continue to experience a greater burden of ill health and lower life expectancy than non-Indigenous Australians [1]. Diet and food insecurity are inter-related and major contributors to the disproportionate burden of disease and premature deaths borne by First Nations Peoples in Australia [2, 3] and in other high-income, colonised countries (Canada, New Zealand and the United States of America) [4].

Food security is defined by the Food and Agriculture Organization as when all people at all times have “physical and economic access to sufficient, safe and nutritious food to meet their dietary needs and food preferences for an active and healthy life” [5]. Food security is determined by availability, accessibility, affordability and acceptability of food.

Being food secure means not just having a sufficient *quantity* of food; it is having access – both physical and economic – to *quality*, “safe and nutritious”, food [5]. It implies that people “have sufficient money to purchase the food they want to eat, to meet cultural and social as well as health and nutritional norms; that this money is not absorbed in other expenditure demands (rent, fuel, debt repayment, etc.); [and] that people can … obtain food in ways which are dignified and in keeping with social norms” [6].

The (un)affordability of healthy diets is the most common barrier to improved nutrition reported by Aboriginal and Torres Strait Islander peoples [7]. For Aboriginal and Torres Strait Islander families in both urban [8] and remote [9] areas, food affordability, related to both income and living expenses, is a major barrier to a healthy diet. Particularly in remote communities, where food prices are higher and median incomes are lower than urban areas, a greater percentage of the household income is spent on food, and poverty impacts negatively on food options [9, 10].

While there is a significant body of literature exploring sociodemographic correlates and other determinants of food insecurity in First Nations populations, there is limited empirical research focused on efforts to mitigate these factors [4]. In particular, interventions addressing economic access to healthy food – either by reducing the cost of healthy food available or increasing household resources and income to purchase healthy food – have been limited and few evaluations had been published [11]. In Australia [12, 13] and other high-income countries [14, 15] store-based and supply chain interventions have dominated efforts to improve food insecurity and nutrition-related outcomes with First Nations Peoples. There is a need to better understand how to tackle the factors that drive persistent socio-economic inequities and poverty [16], and particularly to examine policy responses to improve economic access to healthy foods and diets [17, 18]. This literature review was commissioned by the Ngaanyatjarra Pitjantjatjara Yankunytjatjara Womens’ Council (NPYWC) to inform their continued efforts to improve food security in the remote Aboriginal communities they service in Central Australia, particularly on the Anangu Pitjantjatjara Yankunytjatjara (APY) Lands in Central Australia [9, 19].

The aim of the systematic scoping review of the literature was to identify interventions and any evaluations to improve economic access to healthy foods for First Nations Peoples, that could be considered for application by the NPYWC in the APY Lands.

The primary research question was:

*What interventions addressing economic access to healthy food have been implemented in Aboriginal and Torres Strait Islander communities in Australia, and First Nations communities in other selected high-income, colonised countries (Canada, New Zealand, and the United States of America)?*

The secondary research question was:

*For identified interventions that had been evaluated, what worked and why, and what did not work and why not?*

## Methods

Systematic searches and data extraction were conducted in accordance with the guidance of the Preferred Reporting Items for Systematic Reviews and Meta-Analyses (PRISMA) statement [20].

### Search strategy

The search strategy aimed to identify peer-reviewed articles and other published documents reporting an assessment, case study of intervention/s or policy/ies, or evaluation aimed at directly or indirectly improving economic access to healthy food (alone or among other dimensions of food security).

To develop the research questions and search strategy we used the PICOT (population, intervention, comparator, outcome and timeframe) framework:

**P** = Aboriginal and Torres Strait Islander peoples, and First Nations Peoples in other selected high-income, colonised countries (Canada, New Zealand, United States)

**I** = policy or intervention to improve economic access to food security

**C** = no policy/intervention

**O** = food security or improved affordability of healthy diet

**T** = 1996^1^ to May 2022 (inclusive).

Eight online databases (Table 1) were systematically searched using a combination of four sets of keywords related to:

1. economic access component of food security/affordability
2. First Nations populations
3. country setting
4. policy or intervention.

**Table 1:** Databases and websites searched

Databases	Websites
PubMed	Australian Institute of Aboriginal and Torres Strait Islander Studies
Web of Science	Australian Indigenous Health InfoNet
Cochrane Library	Australian Government Department of Health
Econlit	National Indigenous Australians Agency
Social Science	Indigenous.gov.au
Informit Indigenous collection (INFORMIT)	Australian Institute of Family Studies
Australian Public Affairs (APAFT) (INFORMIT)	Centre for Aboriginal Economic Policy Research (Australia)
ATSIHEALTH (INFORMIT)	Indigenous studies portal research tool (iPortal) (Canada)
	National Collaborating Centre for Indigenous Health (Canada)
	Government of Canada
	Indigenous Services Canada
	Nutrition North Canada
	PROOF (Food Insecurity Policy Research program) (Canada)
	Food Secure Canada
	US Economic Research Service (US Department of Agriculture)
	US Food and Nutrition Service (US Department of Agriculture)
	US First Nations Development Institute
	US National Institute of Food and Agriculture
	NZ Ministry of Health - Maori Health
	NZ Ministry of Health
	The Hub (repository for NZ Government social science research)
	Google

Search terms used are listed in Table 2. These search terms were developed based on previous reviews with similar foci and researchers’ a priori knowledge and refined through an iterative process including test searches in PubMed. An example of the detailed search strategy (PubMed) is included at Supplementary File 1. Terms for First Nations populations are those most commonly used in English language academic literature and per The Lancet-Lowitja Institute Global Collaboration on Indigenous and tribal peoples’ health [21]. We respectfully acknowledge that some tribal and First Nations groups may use or prefer other nomenclature or terminology, and hand searched for such terms in the bibliographies of relevant papers identified.

**Table 2:** Search terms

Search term groups	Keywords
**Economic access component of food security/affordability**	1. food security [MeSH] 2. “food secur*” OR “food insecur*” OR “food sufficien*” OR “food insufficien*” OR “food access*” OR “food afford*” OR “food sovereign*” OR “food pric*” OR “food subsid*” 3. (diet OR fruit OR vegetable OR grocer* OR nutrition* OR meal) AND (afford* OR pric* OR access*)
**Intervention terms**	4. intervention OR policy OR policies OR strateg* OR evaluat* 5. income OR “cost of living” OR poverty OR financ* OR budget* OR payment OR benefit OR money OR cash OR supplement* OR voucher OR coupon OR expen* OR spend* OR purchas* OR buy OR subsid* OR welfare OR “social security” OR “social support” OR “social protection” OR “social enterprise” OR tax OR taxation
**Population groups**	6. Aborigin* OR Torres Strait Island* OR Indigen* OR “First Nation*” OR Maori OR Inuit OR Metis OR “Native Canadian” OR “Native American” OR “American Indian” OR “Alaska Native” OR “first people*” OR “native group*”
**Included countries**	7. Australia* OR “New Zealand” OR “NZ” OR Canada OR “United States” OR “US” OR “USA” OR “North America”
**Combined searches**	8. 1 OR 2 OR 3 9. 8 AND 4 AND 5 AND 6 AND 7 10. 9 + Filters: NOT animal; publication date: 01/01/1996 to present

Websites and research hubs of relevant organisations and agencies (known to the authors or identified from Google searches or other reviews; listed in Table 1) were searched using the site’s database or search tool using combinations of the keywords, depending on the site content. The first 50 returns, or all returns if less than 50, were screened. Several search queries combining terms were conducted using Google and the first five pages of returns (equivalent to 50 returns) were screened.

All searches were conducted between May and August 2022. Backward and forward reference searches also were conducted: reference lists of included studies and previous systematic reviews were hand searched, and we also searched for more recent articles citing particularly relevant articles.

### Study selection

**Fig. 1:**
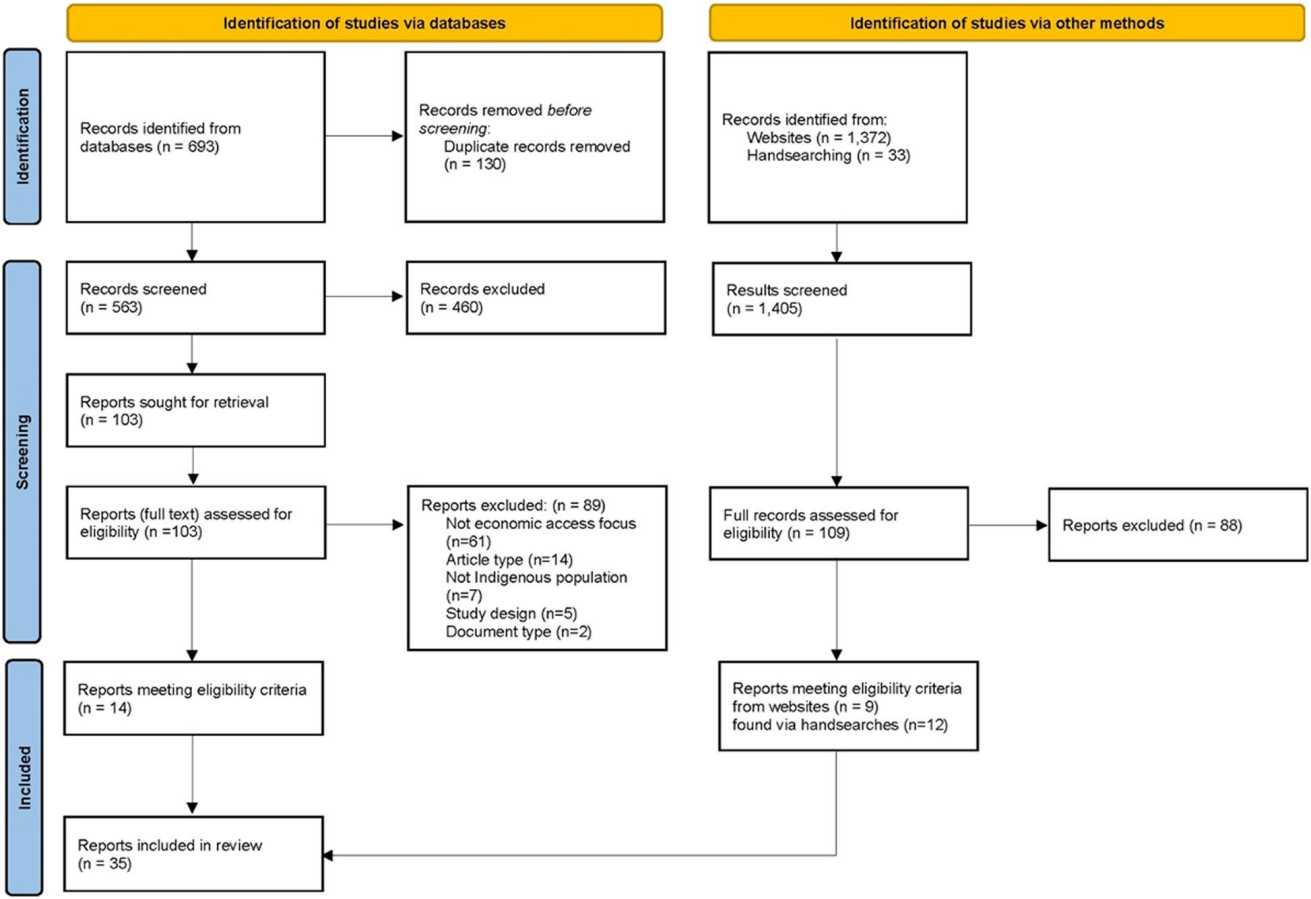
PRISMA flowchart of identification, screening and assessment of studies

The PRISMA flow diagram (Fig. 1) depicts the screening and study selection process.

Database search results were uploaded to Covidence [22] for screening. After duplicates were identified and removed (126 detected by Covidence and four by LH), titles and abstracts of articles were independently screened by two researchers (LH and AL) with reference to the inclusion and exclusion criteria (Table 3); differences were resolved by discussion. The full text of remaining articles was retrieved and reviewed.

**Table 3:** Eligibility criteria for study inclusion or exclusion

Criteria	Include	Exclude
Population	First Nations Peoples/communities in included countries	Non-Indigenous population
Setting	Australia, Canada, New Zealand, United States of America	All other countries
Article type	Original research Systematic review of studies	Abstract Comment or editorial Study protocol or methods paper Narrative review
Study focus	Assessment, case study of intervention/s or policy/s, or evaluation aimed at directly or indirectly improving economic access to healthy food (alone or among other dimensions of food security)	Description of an intervention or policy that was neither implemented nor evaluated. Description or assessment of diet, food security and/or health outcomes unrelated to economic access to healthy food
Study design	Meta-analysis Systematic review of studies Randomised controlled trial (RCT) Interrupted time series Cohort Cost-effectiveness modelling Secondary analysis of data Case study	Cross-sectional Observational
Research methods	Qualitative Quantitative Multiple and mixed methods	
Year of publication	1996-2022	Before 1996
Language	English	Language other than English
Document type	Peer-reviewed journal articles Evaluation report Report of program review	Unpublished articles Thesis or dissertation Book, book chapter Blog News item Media release

Website returns were screened by assessing the page title and accompanying description and/or first screen (webpage or document) for potential relevancy. Web addresses (URLs) of potentially relevant records were copied to an Excel spreadsheet for full text review.

Separate articles reporting on the same study were included if they reported different findings relevant to this review’s aim; earlier articles were omitted if they reported interim or preliminary results and final results were reported in a subsequent publication that was included.

### Data extraction

Data from the included articles were extracted to a spreadsheet with agreed fields (Supplementary File 2) by one researcher (LH) and checked by a second (AL). Consistent with the a priori objectives of the review, only data relevant to economic access healthy food were extracted. In addition to study details (year published, setting, population and study design) these included the intervention (or study) aim; details of the policy or intervention; the lead agency (e.g. government, community, tribal-University partnership); data and measures; results of process, impact and outcome evaluation/s; identified barriers to and/or enablers of effectiveness of the policy/intervention; and the authors’ recommendations relevant to the aims of this review.

### Quality assessment

As the aim of this search was to identify any evidence from any intervention, evaluation or assessment of an intervention or policy that might be effective in improving economic access to healthy food for First Nations Peoples in the selected countries, it was undesirable to limit study inclusion on the basis of the quality of the studies. While searches were conducted systematically, in this regard the review was consistent with comprehensive scoping reviews [23].

### Data synthesis

As most of the data in included studies were qualitative, a descriptive synthesis was conducted to collate findings (Supplementary File 2). Key barriers to and enablers of successful implementation and/or impacts of the interventions were identified during data analysis and synthesis using the constant comparative method, in which the data were sorted into groups and organised by key attributes [24].

### Data interpretation

The data synthesis was presented to a workshop with the NPYWC Anangu research team in Alice Springs, Central Australia on 28-29 November 2022. Results were reviewed and discussed in traditional ‘yarning’ style [9] by all participants with the aim of identifying relevant interventions with potential merit for application on the APY Lands.

## Results

The multiple search strategies yielded 2098 potentially relevant records for screening (Fig. 1). After screening the title and abstract of records from database searches, 103 articles were retrieved for full text review. After screening results of website searches and hand searches, 109 full records were assessed for eligibility. The main reason articles and reports were excluded was that they were not an assessment, case study or evaluation of a policy or intervention/s addressing economic access to healthy food (Fig. 1). After scrutiny and assessment, 35 papers/reports met the eligibility criteria and were included.

### Study characteristics

Detailed data extraction spreadsheets have been provided as Supplementary File 2. Data from the included papers and reports were synthesised by the type of intervention/policy implemented (Table 4). The key characteristics of the included studies are summarised in Table 5.

**Table 5:** Key characteristics of included studies

Characteristic	Number of publications (total *n* = 35)	Number of discrete interventions (total *n* = 21)
Country setting		
Australia	13	8
United States of America	11	9
Canada	9	3
New Zealand	2	1
Type of intervention		
1. Price discount on healthy food	**13**	**7**
a. sold in community store	10	5
b. sold via mobile grocery van	1	1
c. provided by health service	2	1
2. Subsidies to retail stores, suppliers and producers	**8**	**2**
a. Direct subsidies to retail stores	5	1
b.Transport subsidies	3	1
3. Free healthy foods and/or vouchers provided to community members	**8**	**7**
a. Healthy food provided	5^a^	4
b. Vouchers redeemable for healthy food	4^a^	4
4. Increased income (for food purchases)	**1**	**1**
5. Government strategies/policies (not otherwise described above)	**5**	**4**
a. Tax waiver on healthy foods	1	1
b. National Food Security Strategy	1	1
c. Income management	2	1
d. Community economic development	1	1

^a^ One intervention [46] provided monthly food box of shelf-stable nutritious foods *and* a voucher redeemable for F&V

Of the 35 included papers/reports (Supplementary File 2, Table 4) the greatest proportion were set in Australia (*n* = 13, 37%) with 11 in the US, nine in Canada and two in New Zealand. Several papers/reports considered the same intervention, hence only 21 discrete interventions were identified (Table 5). Most of the studies were set in, or related to, rural and remote communities. Most targeted healthy foods, and some focused on fruit and vegetables specifically; few targeted unhealthy foods (Table 4).

**Table 4:** Synthesis of data from included studies, categorised by the type of intervention/policy implemented

Study reference	Setting	Intervention dose/policy details	Nutrition education provided also	Process, Impact, Outcome [measures]	Economic measure?	Evaluation findings	Identified barriers	Identified enablers	Specific recommendations (of study authors)	Promising	Comments/Notes
**1. Price discount on healthy food sold via retail stores, mobile grocery van, or health service (*n*** **= 13 publications,** ***n*** **= 7 studies)**											
**1a Provided by community retail store (*n*** **= 10 publications,** ***n*** **= 5 studies)**											
Williams, 2021 [25]	USA: Chickasaw and Choctaw nations, Oklahoma	“THRIVE”: Cluster-controlled trial (in 2 Nations; in each 2 stores received intervention, 2 were controls; 9 months in Nation A; 12 months in Nation B); offered healthy ready-to-eat meals and snacks (high in F&V) at or below prices of competing foods.	N	Weekly sales data (first 6 months of intervention)	N	F&V basket sales higher in intervention stores than controls (significantly higher in one nation, but not the other); total sales remained steady.	A	ABCE	Regular updated promotions needed	Y	
Blue Bird Jernigan, 2019 [26]	USA: Chickasaw and Choctaw nations, Oklahoma	“THRIVE”: Cluster-controlled trial (*n* = 1204 in 2 Nations; 9 months in Nation A; 12 months in Nation B); offered ready-to-eat healthy meals and snacks (high in F&V) at or below prices of competing foods.	N	Self-reported dietary intake; recall of promotions and reported purchasing	N	Increased purchasing of fruit, vegetables and other healthy foods; however, F&V intake did not increase in either Nation.		ABE		Y	Discounted ready-to-eat healthy meals/snacks of high relevance to APY communities
Brimblecombe, 2018 [27]	Australia; Northern Territory	“SHOP@RIC” trial: Price discount (20%) on fresh and frozen fruit and vegetables, bottled water and artificially sweetened soft drinks +/− consumer education. Stepped-wedge RCT in 20 very remote communities; *n* = 148 adults who identified as primary shopper for household. 49 week baseline data-collection, then 24 week intervention, 24 week post intervention follow-up.	Y +/−	148 adults. Self-reported intake; mediators and moderators	N	Modified perceived affordability of F&V but no substantial consumer behaviour change.	B		Long-term government investment and commitment needed to address underlying constraints, including monetary incentives; need to enhance self-efficacy to cook and try new vegetables.	Maybe, if identified barriers addressed	Discount was not strong enough to overcome constraints in those most disadvantaged. No consideration of social response bias.
Magnus, 2018 [28]	Australia: Northern Territory	Estimated cost-effectiveness of 20% price discount on healthy food and beverages (+/− nutrition education); analysis alongside the SHOP@RIC trial (above) in 20 remote communities.	Y +/−	Food sales data; published mortality, disease and RF data; costs and cost-offsets	Y	20% discount with or without consumer education cost more money without leading to health gain, i.e. it offered poor value for money	B			Maybe, if identified limitations addressed	
Brimblecombe, 2017 [29]	Australia: Northern Territory	Price discount (20%) on fresh and frozen fruit and vegetables, bottled water and artificially sweetened soft drinks +/− consumer education (SHOP@RIC trial); stepped-wedge RCT in 20 very remote communities; 49 week baseline data-collection, then 24 week intervention, 24 week post intervention follow-up.	Y +/−	Weekly store sales data	N	Complete implementation of discount promotion and consumer education not achieved in all stores. Positive shift in purchases of F&V and bottled water but not diet drinks. Price discount alone was associated with a 12.7% increase in purchases in grams of fruit and vegetables combined (primary outcome); and a 19.8% increase after discount had ceased (after vs before). Purchases of water and diet and regular soft drinks also increased post-intervention.	BJM	B	Price discount on healthy foods may need to be supported by price increase of unhealthy foods; greater promotion of F&V; cooking and food budgeting programs; improved household food preparation and storage infrastructure; and education to discourage unhealthy choices.	Maybe, if identified limitations addressed	Possible unintended consequences, with cost savings lead to increased consumption of unhealthy products
Magnus, 2016 [30]	Australia: Northern Territory	Modelling estimated cost effectiveness of six price discount strategies on fruit, vegetables, diet drinks and water	Y +/−	Food sales data; published price elasticity data; Aboriginal population health status indicators	Y	All fiscal strategies modelled had positive impact on diet quality; 5/6 estimated as cost effective (below $50,000/DALY threshold)			Price discounts appear to be potentially cost-effective	Maybe, if similar results after implementation in the real world	Need to consider difficulties around implementation and impact on magnitude
Ferguson, 2017 [31]	Australia: 18 remote communities in the Northern Territory and Western Australia	Four price discount strategies: reduce markup on grocery products; fresh F&V point-of-sale scales; fresh F&V at landed cost; diet soft-drink discount. Implemented since 2010. *N* = 54 informants	N	Retrospective evaluation of a natural experiment.	N	Discounts applied as intended; varying levels of promotional materials. No effect of price discount (10%). Non-significant reduction of diet soft drinks	B	ABC	Greater dose, duration and promotion of discounts; co-design including customers, store owners and staff; monitoring of implementation	Maybe, if identified limitations addressed	
Lee, 2016 [19]	Australia: remote South Australia (APY Lands)	Store nutrition policies and interventions to address healthy food availability, accessibility and affordability; time series of cross-sectional studies. *N* = 7 communities	N	Food price and availability and sales data	N	Decreased price of F&V. Since 1986, cross-subsidisation increased availability and affordability of healthy foods, especially F&V. Increased supply and intake of discretionary foods, too,	L	ACE	Sustained community effort needed to improve availability and affordability of healthy food.	Maybe, if identified limitations addressed	Mai Wiru store policy needs update/revision. Suggested more frequent monitoring of stores and increased engagement of all community members in results
Blakely, 2011 [32]	New Zealand	“SHOP” RCT: Assessment to determine if effects of price discounts of 12.5% on healthy foods varied by ethnicity, income or educational qualifications.	Y +/−	Purchasing data (barcode scanner)	N	Price discounts had a weaker and null effect among Māori than among European New Zealanders	BI		Better targeting could be warranted	Maybe, if identified limitations addressed	
Ni Mhurchu, 2010 [33]	New Zealand	“SHOP” RCT: Price discounts of 12.5% on healthy foods applied automatically at checkout +/− tailored nutrition education; information on price discounts mailed to participants. 12-week baseline; 24 week intervention; 24 week follow-up; *n* = 1104 adult shoppers at 8 supermarkets (23% Māori).	Y +/−	Pre- and post- data. Purchasing data (barcode scanner)	N	Healthy food purchases improved slightly with price discounts but no significant improvement in nutrient analysis; price discounts had sustained but small effect on F&V purchases; education had no effect on food purchases.	BHI		In-store signage and ‘shelf-talkers’ would be better promotional tools than list of products eligible for discount.	Maybe, if identified limitations addressed	
**1b Provided via mobile grocery van (*n*- = 1 paper,** ***n*** **= 1 study)**											
Cueva, 2018 [34]	USA (an unnamed Native American community)	Mobile grocery (Mo Gro) offering subsidised healthy food twice a week; 3-month evaluation; *n* = 92 First Nations households (randomised selection of 20% of households).	Y	Self-reported food purchasing, consumption and perceptions; FS questionnaire	N	Process: Served avg. of 71 customers per visit (twice weekly); Impact: 75% reported change in food purchases, 68% changed dietary patterns; Outcomes: FI declined from 57 to 43%		ABE	Need to include traditional foods	Y	Developed in response to community need assessment
**1c provided via health service (*n*** **= 2 papers;** ***n*** **= 1 study)**											
Black, 2014 [35]	Australia: rural NSW communities	Subsidised F&V ($5 for box of $40 value, or $60 if 5 or more children); also in one community vouchers redeemable at F&V shop; 55 low-income families, 121 participating children; duration of program highly variable (several features mimicked a natural experiment).	N	Pre- and post- of varied duration. 24-hour dietary recall and biomarkers	N	70% of families collected 75% or more of available F&V boxes; improved biomarkers in children but not changes in self-reported intake of F&V	D		Controlled study of subsidised healthy foods is warranted	Maybe, if identified limitations addressed	Occurred in real world setting so difficult to account for all potential confounders.
Black, 2013 [36]	Australia: rural NSW communities	Subsidised F&V, as above	N	Health service use; biomarkers and child height and weight	N	Decreased presentations to health clinic	D			Maybe, if identified limitations addressed	
**2. Subsidies to retail stores, suppliers and producers (*n*** **= 8, studies = 2)**											
**2a Subsidies direct to retail stores (*n*** **= 5 papers/reports,** ***n*** **= 1 study)**											
CIRNAC, 2020 [37]	Canada: Remote, northern communities	Nutrition North Canada: government subsidy provided directly to (contracted) retailers, suppliers and registered country food processors to reduce costs of nutritious perishable foods for residents of remote northern communities (subsidy rates vary depending on type of transportation used, location of the community, and category of eligible food and non-food items); horizontal evaluation. Program commenced in 2011.	N	Qual: interviews, document review, analysis of program data	N	Low population awareness of program and understanding of how subsidy works; increased access to nutritious perishable food at subsidised rate but subsidy has minimal impact on affordability, especially for people on low income (welfare or minimum wage) and seniors; recommended diet still unaffordable (typical household of four able to afford less than half contents of recommended food basket); some staple items not subsidised e.g. flour and lard; minimal savings often perceived negatively by community.	BCIM	E	Need to work better with communities; increase magnitude/dose; develop indicators that are relevant to CPI; include subsidies for local food production; improve promotion of program.	Maybe, if identified limitations addressed	Subsidies at point-of-sale or directly to vulnerable consumers could be more effective
Naylor, 2020 [38]	Canada: Remote northern communities	Nutrition North Canada; econometric assessment of pass-through rate through food supply system ($ input v benefit)	N	Published food price data	Y	Subsidy appears to reduce food prices i.e. dollar increase in subsidy is associated with a dollar reduction in final food price; higher pass-through rates in larger communities due to economies of scale and density in air transportation; subsidised food items relatively cheaper than in Ottawa.		E	Increase amount of subsidy provided, and target subsidy to specific food items desired by FI households	Maybe, if identified limitations addressed	Number of eligible communities varied
St-Germain, 2019 [39]	Canada: Remote northern communities	Nutrition North Canada; interrupted time series regression analysis (*n* = 3250 households in 10 communities)	N	Self-reported food insecurity	N	Prevalence of household food insecurity increased from 33.1% in 2010 (year before launch), to 39.4% in 2011 (year of launch) and 46.6% in 2014 (year after full implementation)	C	E	Subsidy on nutritious food only could increase food security for the most economically vulnerable households. More research is needed to investigate food access inequality.	Maybe, if identified limitations addressed	
Galloway, 2017 [40]	Canada: Remote northern communities	Nutrition North Canada; program evaluation	N	Program data and evaluations; sales and price reports	N	Persistent inequities in food pricing between communities and food items (in absence of price caps) and population groups (e.g. some individuals order directly); subsidies of insufficient magnitude to address inequalities.	BFGJK	DE	Need for increased retailer accountability and regulatory framework.	Maybe, if identified limitations addressed	Retail subsidy not effective where there is not a competitive marketplace
Auditor General of Canada, 2014 [41]	Canada: Remote northern communities	Nutrition North Canada; program audit.	N	Audit.Qual: stakeholder interviews; policy/document analysis	N	Weight of items subsidised increased by about 25% but did not improve FS; lack of transparency in program management	F	E	Need for greater compliance monitoring and requirement for retailers to provide information needed to assess whether they are passing on full subsidy to consumers	Maybe, if identified limitations addressed	
**2b Subsidies for transport (*n*** **= 3 papers/reports,** ***n*** **= 1 study)**											
INAAC, 2009b [42]	Canada: Remote northern communities	Food Mail Program (FMP) - subsidised cost of transporting nutritious perishable food to remote communities; program evaluation.	N	Statistical and econometric analyses; Qual – panels, interviews	Y	Reduced prices of food, but still unaffordable for many households. Increased subsidy rates for priority perishable foods (e.g. vegetables, fruit, eggs) in three pilot project communities resulted in significantly higher per capita volume shipments and presumably consumption of perishable items	BCGJ		Need increased transparency and accountability; to engage with Aboriginal organisations to help ensure items are culturally appropriate; support local, sustainable, complementary initiatives e.g. community freezers; increase subsides on ‘staples’ such as bread and milk to increase affordability.	Maybe, if identified limitations addressed	Concern about degree to which subsidies are passed on to communities. Program ran from the 1960s to 2011 in different formats.
INAC, 2009a [43]	Canada: Remote northern communities	FMP (as above); program review (separate process to above).	N	Program costs; food prices	Y	FMP successful in lowering the price of food in participating communities; further reductions in shipping rates for “priority perishable foods” (in pilot project) resulted in price reductions of about 15 to 20%.			Need increased transparency and accountability of retailers; investigate redesign of program as a retail subsidy delivering benefits to consumers at point of purchase.	Maybe, if identified limitations addressed	
Dargo, 2008 [44]	Canada: Remote northern communities	FMP (as above); independent review.	N	Program data; discussion with stakeholders	N	Poor program evaluation processes; program burdened with “design, logistical, administrative, accountability, negative resident perception and application issues”; Low levels of awareness; many residents concerned subsidy was not being passed on.	FHI		Replace with new program providing better subsidy on core basic items, developed in partnership with Inuit organisations	Maybe, if identified limitations addressed	
**3. Provision of healthy food (*n*** **= 8 reports/papers,** ***n*** **= 7 studies)**											
**3a Healthy food provided at no cost (*n*** **= 5 reports/papers,** ***n*** **= 4 studies)**											
Ahmed, 2020 [45]	USA: Rural; Flathead reservation, Montana	Pilot study of “Eat Fresh”; weekly boxes of recommended servings of fresh fruits and vegetables provided for six weeks; *n* = 19 low-income Native American adults	Y	Pre- and post- intervention. Qual: diet habits and health perception; Quant: diet intake, biomarkers	N	Reported increase in F&V variety; trend of improved diet quality; significant HEI increase post-intervention; BMI and blood pressure increased	CDM	A	Need for multi-strategy, holistic dietary interventions and focus on whole diet; should measure multiple indicators, both qualitative and quantitative; important to collaborate with Community Advisory Board for intervention design and feedback.	N	Small study; objective outcomes worsened
Briefel, 2021 [also at 3b] [46]	USA: Chickasaw Nation	Monthly food box (shelf-stable nutritious foods) and $15 voucher for F&V for each eligible child; cluster RCT in 40 school districts in Chickasaw Nation over 25 months (*n* = 2859, 14% Native American)		Food security (survey), food expenditure	Y	Participation rate 61% (boxes had to be ordered online or by phone); did not improve child FS; adult FS improved initially but not at follow up; modest decline in out-of-pocket food expenditure	F			Y	Several confounders including improved economic circumstances of the population and participation in other nutrition assistance programs
Pindus, 2019 [47]	USA: Rural/remote and urban reservations in the Klamath River Basin	Review of the Food Distribution Program on Indian Reservations (FDPIR), providing monthly food packages (perishable and non-perishable) to low-income households living on Indian reservations, on tribal lands, and other designated areas (*n* = 1053 households). Duration not clear.	Y (variable)	Participation; Qual: FS measures, discussion groups	N	FDPIR was only source of food for 38% of participants; 34% of households had low FS and 22% continued to have very low FS. Food package was inadequate in rural areas; not meeting community needs.	EFH		Establish partnerships, and expand supplemental assistance and/or food access and flexibility	N	Government program - no mention of co-design
Mucioki, 2018 [48]	USA: Rural/remote and urban reservations in the Klamath River Basin	Case study. FDPIR (as described above). *n* = 151 using FDPIR, 275 using other food assistance, 242 not using food assistance	Y (variable)	Qual: perceptions and operations via interviews and focus groups; FS measures	N	Packages do not meet international standards for quality, access, availability, nutrition and cultural appropriateness. Participants desire more fresh fruit, vegetables and traditional foods. Food boxes are essential source of food, but fail to alleviate FI.	EF		Increase amount and frequency of delivery of F&V; support traditional food acquisition; increase eligibility	N	
Ichumar, 2018 [49]	Australia: Rural Western Australia	School breakfast program; in two schools with high Aboriginal student populations; duration not clear	Y	Stakeholder interviews, observation, document review	N	Food provided passively to children, not necessarily nutritious; little evidence of health education	F		Schools should explore arrangements with local growers/shop owners with respect to support for the SBP.	N	
**3b Healthy food vouchers (*n*** **= 4 reports/papers,** ***n*** **= 4 studies)**											
Briefel, 2021 [also at 3a] [46]	USA	See above ($15 voucher for F&V for each eligible child provided with monthly food box)	*As reported above*								
Jones, 2020 [50]	USA: Navajo Nation	Fruit and vegetable prescription (FVRx): vouchers redeemable for fruit, vegetables and healthy traditional foods from participating retailers; US$1 per household member per day, with a maximum value of $5/day; 243 Navajo children. Ran May 2015 to Sept 2018.	Y	F&V consumption and food security, child height and weight	N	Process: 65% of children retained in program > 6 months; Outcomes: household FS increased from 18 to 35%		CE	–	Y	Multiple confounders - difficult to isolate or attribute outcomes
McLaury, 2016 [51]	USA: Rural reservations in Washington State	Cash value vouchers (CVV) for F&V added to WIC food packages (monthly values of $6 for children and $10 for pregnant, breastfeeding, and postpartum women). Duration not clear.	N	Program data (vouchers issued and redeemed)	N	No significant outcomes in American Indian population	F		More research needed to determine causes of low voucher redemption, including socioeconomic and cultural barriers to CVV redemption on reservations.	N	Authors presume barriers such as embarrassment, unfamiliarity with F&V, cost of produce, misunderstandings about how to use vouchers
Brown, 2019 [52]	Australia: Remote communities, Cape York, Queensland	F&V voucher; 32 weeks over two phases (1: $10 voucher for minimum $20 spend on F&V; 2: $10 voucher for minimum $15 spend). Pre and post; impact measure was F&V purchasing.	Y (ad hoc)	Pre- and post- Qual interviews; store sales data	N	Trend of reduced F&V sales and overall food and drink; 7% reduction in fruit sales. Average voucher redemption rate was 29%. Highest use of vouchers (44%) in week when project staff promoted program/cooking demonstrations in store.	GM	BC	Target vouchers to women and children; use store loyalty cards instead of paper vouchers; increase flexibility of redemption (greater variety of healthy foods); increase promotion; need more support from store staff.	Maybe, if identified limitations addressed	Not clear how controlled for community numbers/store population. Precursor to study by Ferguson et al. (2017).
**4. Provision of greater income to community members (*n*** **= 1 paper,** ***n*** **= 1 study)**											
Gordon, 2017 [53]	USA: 14 sites including two tribal nations (Cherokee and Chickasaw)	Piloted “Summer Electronic Benefit Transfers for Children” (SEBTC), cash benefit of $60/child/summer month; *n* = 42,000 households in 14 sites, 2 tribal nations, duration = one summer period	N	Food frequency questionnaire and food security scale	N	Significantly reduced rates of very low FS (one-third lower for households receiving SEBTC). Children in households receiving benefits consumed more healthy foods including F&V. Impacts in WIC sites were at least twice as large as those in SNAP sites (where benefits could be used to purchase SSBs; WIC-model restricted to healthy food).		E	Model deserves consideration; providing benefits in summer meets gap for children who receive school-based nutrition programs during school terms.	Y	
**5. Government strategy/policy (not otherwise described above) (*n*** **= 5 reports/papers;** ***n*** **= 4 studies)**											
George, 2021 [54]	USA: Navajo Nation and bordering towns	Tax Waiver (healthy foods): Hypothecated tax on unhealthy foods in Navajo Nation (2%; revenue directed to local community wellness projects) and waiver of sales tax on healthy items (including water, fresh fruits and vegetables and nuts).	N	Store surveys in 2013 and 2019 (matched sample of 71 stores: 51 in Navajo Nation, 20 in border towns)	N	Since 2013 (after adjusting for inflation), average cost per item of fresh fruit decreased by 13% in Navajo stores and increased in border stores, resulting in comparable prices in Navajo and border stores in 2019. Pricing trends among vegetables and other healthy foods were inconsistent.	B			Maybe, if identified limitations addressed	Only measured changes in pricing and food availability (and in-store promotion).
ANAO, 2014 [55]	Australia: National, focus on remote communities	National Food Security Strategy: Australian Government implementation of food security initiatives under ‘Close the Gap’ for remote Indigenous communities including targeting affordability.	Y	Performance audit	N	Pilot sites identified. However, there was no evidence that initiatives to decrease the prices of healthy foods had been implemented.	G		Strategy required a funded action plan, and implementation.	Maybe, if identified limitations addressed	Throughout Australia “basic, healthy foods” do not incur 10% GST. Australian Government currently developing another Remote Indigenous community food supply strategy.
Bray, 2014 [56]	Australia: Northern Territory	Income Management: Northern Territory (NT) New Income Management policy, operationalised through use of EFTPOS card (“BasicsCard”) able to be used only in approved stores and not to purchase prohibited goods (e.g. alcohol) or withdraw cash. Intervention commenced in 2007. Policy evaluation.	N	Longitudinal survey; store transactions	N	No evidence of changes in spending patterns, including food and alcohol sales, other than a slight possible improvement in the incidence of running out of money for food by those on Voluntary Income Management, but no change for those on compulsory income management.	HKM		Program should be voluntary	N	
Brimblecombe, 2010 [27]	Australia: 10 remote communities in the Northern Territory with stores managed by Arnhem Land Progress Association.	Income management policy: 50% of income support and family assistance payments (and 100% of lump sum payments) to Indigenous people living in remote areas of the NT to be used only for items considered essential by the government, such as food, clothes, rent, etc. Analysis of sales data from 10 community stores over 3 years (October 2006 to September 2009).	N	Interrupted time series analysis: store sales before (18-month period) and after introduction (4-6 month period) of income management, 3 months coinciding with a government stimulus payment, and remaining income-management period	N	Income management had no effect on fruit and vegetable sales or turnover; significant increase in sales (total store, total food and beverage, fruit and vegetable and soft drink) during period of government stimulus payment.	HKM		Unintended consequences with increased sales of sugar sweetened beverages.	N	Limited products studied
Thompson, 2012 [57]	Canada: Rural Northern Manitoba	Country food programs that support people living off the land to feed the local community; participatory process over four years; analysis of 7 communities with “best practice” in food programming and 7 with limited uptake. Random selection of 553 households. Duration of program unclear.	N	Food costs and food security surveys (553 households in 14 rural communities)	N	Country food programs were related to better food security; food programs that enable sharing of traditional foods improved food security more than other variables, such as access to stores.		A	Improving food access requires community control over funding and decision-making without undue restrictions on country foods.	Y, where sustainable traditional foods available	May not apply in all First Nations communities, especially where traditional food systems are under threat by climate change, population pressure etc.

**Identified barriers**: A: Duration. B: Magnitude or dose. C: Lack of economic access to other foods and essential items. D: Only fruit and vegetables. E: Predominantly long shelf-life foods. F: Poor distribution, access issues/inequities. G: Store issues and compliance (e.g., staff training, high staff turnover). H: Poor targeting of population. I: Lack of promotion or awareness. J: Market price fluctuations and retail pricing practices. K: Lack of retail competition. L: External pressures of global food system. M: No time, health hardware, or resources to cook.

**Identified enablers**: A: Community control/empowerment and/or co-design. B: Program well promoted. C: Retail support via store infrastructure or nutrition policy. D: Opportunity cost considered. E: Focus on all healthy foods.

Abbreviations/symbols that might need explaining: *Y* yes, N no, *Y +/−* sometimes; either as RCT or ad hoc, *F&V* fruits and vegetables, *FI* Food insecurity, *FS* Food security, *RCT* randomised controlled trial, *SNAP* Supplemental Nutrition Assistance Program, *SSBs* sugar-sweetened beverages, *WIC* Special Supplemental Nutrition Program for Women, Infants, and Children.

Interventions were classified into five main categories (Table 5):

1. price discounts on healthy food sold in communities;
2. subsidies provided directly to community retail stores, suppliers and producers;
3. free healthy food and/or food vouchers for healthy foods provided to community members;
4. increased income provided to community members (for food purchases); and
5. government strategies and policies (not otherwise described above).

A variety of metrics was used to inform process, impact and/or outcome evaluation, with highly heterogenous results (Supplementary File 2; Table 4). Mixed method evaluations were common, but most evaluations collected qualitative, rather than quantitative, data. Few economic evaluations were conducted. Where provided as part of the intervention, no study attempted to apportion the impact of nutrition education on results specifically. Several studies identified barriers and enablers to effective intervention (Table 4).

## Results of included studies by type of intervention/policy

### Price discount on healthy foods

The most common type of specific intervention was discounting the price of healthy foods sold in communities, with 13 published studies of seven interventions [19, 25–36]. Most papers (*n* = 10) related to five different interventions, which provided price discounts through community retail stores. In one study from the USA, discounted healthy food and drinks were offered for sale via a mobile grocery van [34], and in one Australian study discounted fruit and vegetable boxes were distributed via community health clinics [35, 36].

#### Discounted healthy food in retail stores

Two papers described the implementation and review of the “THRIVE” cluster-controlled trial in the USA, which offered healthy, ready-made meals and snacks at or below prices of competing foods in community stores in two Nations [25, 26]. The evaluation, informed by weekly sales data in the first 6 months [25] and reported dietary intake and recall of promotions and reported purchasing [26], showed increased purchasing of fruit, vegetables and other healthy foods in one Nation, but not the other [25]. However, reported fruit and vegetable intake did not increase in either Nation [26]. Community empowerment, promotion of the program and availability of convenient healthy meals were considered key to success.

As a natural experiment in Australia, Ferguson and colleagues [31] retrospectively evaluated implementation of four food price discount strategies (reduced markup on healthy grocery products, introduction of point-of-sale scales for unpackaged fresh produce, costed fruit and vegetables at landed price; and discounted diet soft drinks) in 18 remote Aboriginal community stores managed by a specific retail group. The study used mixed methods including 54 stakeholder interviews, observation, and historic sales data. Discounts were applied generally as intended; however, no effect of the approximate 10% discount was evident. The authors concluded that impact on food and beverage sales was limited by variable promotion and the limited magnitude of the discount [31].

Also in Australia, four papers described the modelling [30], implementation and evaluation [27, 29] and cost-effectiveness [28] of the “SHOP@RIC” stepped wedge RCT which tested the impact of a price discount of 20% on fresh and frozen fruit and vegetables, bottled water and artificially sweetened soft drinks, with and without nutrition education, in 20 remote communities. The project involved 49-week baseline data-collection, 24-week intervention, and 24-week post intervention follow-up [29]. Analysis of store sales data found complete implementation was not achieved in all stores as planned. However, there was a positive shift in purchase of fruit and vegetable and bottled water, but not diet drinks. Price discount alone was associated with 12.7% increased purchase of fruit and vegetables, and 19.8% increase after discounting ceased. Self-reported dietary intake, mediators and moderators in 148 participants showed improved self-efficacy and perceived affordability of fruit and vegetables but limited dietary change [27]. Economic analysis showed little health gain for the cost of the intervention [28]. The researchers recommended greater dose, duration and promotion of discounts; co-design including customers, store owners and staff; and monitoring throughout implementation. They noted the potential for unintended consequences, and hence that the price discount on healthy foods may have needed to be supported by increasing prices of unhealthy foods. Authors recommended greater promotion of fruit and vegetables, cooking and food budgeting programs, and discouragement of unhealthy choices, and also highlighted the need for political commitment and long term investment to improve household food preparation and storage infrastructure [27, 29].

In another Australian effort to improve food supply in remote Aboriginal communities in Central Australia, cross-subsidisation of healthy foods by increasing the price of unhealthy foods in stores was a component of long-term strategies including development and implementation of a store nutrition policy [19]. Regular surveys of the prices, availability, placement and promotion of healthy and unhealthy foods in stores, along with store sales data, showed prices of fruit and vegetables decreased and intake increased. However, there was also increased supply and intake of unhealthy foods since 1986, mirroring diet changes across broader Australia [19].

In New Zealand, the “SHOP” randomised controlled trial tested application of price discounts of 12.5% on healthy foods at retail store checkouts (promoted to participants by mail), with/without concurrent nutrition education [33]. The 12-week baseline was followed by 24-week intervention and 24-week follow-up in 1104 shoppers (23% were Māori) at eight supermarkets. Healthy food purchases, assessed by bar code analysis of sales, improved slightly with price discounts; however, change in nutritional quality of purchases was not significant. Price discounts had a sustained but small effect on fruit and vegetable purchases; nutrition education had no effect on food purchases. Purchasing data showed no effect among Māori, less than among New Zealanders of European background [32]. The authors recommended more specific targeting and in-store promotion of the discounts [32, 33].

#### Discounted healthy foods provided by mobile grocery van

In the USA, a mobile grocery van (“MoGro”) offered subsidised healthy food and nutrition education to 92 First Nations households [34]. The evaluation was conducted 3 months after implementation, comprising self-reported food purchasing, consumption and perceptions, and administration of a food security questionnaire randomly to 20% of households. Around 71 customers received twice-weekly van visits; of these 75% reported change in food purchases, 68% reported improved dietary patterns, and food insecurity declined from 57 to 43%. Reported strengths of the program were that it was developed in response to community needs assessment, was well promoted, controlled by the communities, and focused on a wide range of healthy foods. Inclusion of more traditional foods was recommended [34].

#### Discounted healthy foods provided through health clinics

In an Australian study involving 55 families in western New South Wales, discounted boxes of fruit and vegetables (of AU$40 or $60 value depending on the number of children in the families) were sold via the health clinic for AU$5 [35, 36]. Seventy percent of families purchased 75% or more of the fruit and vegetable boxes offered during the study. Biomarkers of fruit and vegetable intake improved in the 121 children participating; however self-reported intake of fruit and vegetables did not increase [35, 36]. The authors noted that the study occurred in a real-world setting – for example, different families enrolled in the program at different times – so it was difficult to account for all potential confounders, and recommended an RCT.

### Subsidies to retail stores, suppliers, producers and transporters

The next most commonly described type of intervention was subsidies to retail stores, suppliers and producers either directly (*n* = 5 papers/reports) or by subsidising transport costs (*n* = 3). However, each of these groups of Canadian studies covered the same interventions: the Nutrition North Canada program and the preceding Food Mail Program respectively.

Nutrition North Canada (NNC) was a Canadian Government subsidy provided directly to contracted retailers, suppliers and registered country food processors [37]. Subsidy rates varied depending on location of the community, category of eligible food items, and type of transportation involved. The NNC program was evaluated by analysis of published food price data [38], self-reported food security [39], sales and price data [40], and also audited internally, informed by stakeholder interviews and document analysis [41]. Evaluations found low population awareness of the program and understanding of how the subsidy worked. While some subsidies were passed on, especially in larger communities [38], and access to healthy perishable food at the reduced rates increased, the subsidies had minimal impact on affordability, especially for people on welfare or minimum wage and seniors; hence recommended diets remained unaffordable [37]. The prevalence of household food insecurity increased from 33.1% in 2010 (year before launch), to 39.4% in 2011 (year of launch) and 46.6% in 2014 (year after full implementation) [39]. The subsidies were found to be insufficient in magnitude and the minimal savings were perceived negatively by the communities involved [40, 41]. Recommendations included improved community involvement and promotion, and increased level of subsidisation [37–41].

The Food Mail Program (FMP) in Canada (replaced by the NNC after 2011) subsidised the cost of transporting healthy, perishable food to remote Inuit communities [43]. The program was reviewed twice, informed by food price surveys, cost analysis of program delivery [42] and program data and stakeholder consultation [44]. Although pilot data showed additional reductions in shipping rates for “priority perishable foods” resulted in savings in program delivery costs of 15 to 20% and higher per capita shipment of vegetables, fruit and eggs [44], and that the program lowered the price of food in participating communities, healthy foods were still unaffordable for many households [43]. Further, evaluations reported poor accountability, poor program evaluation design, low levels of awareness, negative resident perceptions, concern that the subsidies were not being passed on to consumers, and need for better engagement with First Nations organisations to identify culturally appropriate healthy foods and infrastructure [42–44].

### Free healthy food or food vouchers for healthy foods

#### Free healthy foods provided to community members/priority groups

Five of the included studies investigated provision of healthy food directly to community members, with a range of products, quantities and frequencies described.

Each month for at least 25 months a box of shelf-stable healthy foods and a US$15 voucher for fruit and vegetables for each eligible child was provided to families participating in a cluster RCT in 40 school districts within the Chickasaw Nation in the USA [46]. Of the 2859 people involved, 14% were Native American [46]. The program was evaluated via food security and food expenditure surveys. Food boxes were ordered online or by phone; the participation rate was 61%. Food security scores of children did not improve; those of adults improved initially, but not at follow up. A modest decline in out-of-pocket food expenditure was found. The results were confounded by changing economic circumstances and resources of the population, and varied participation in other nutrition assistance programs. Poor distribution and access were identified as potential challenges [46].

Two papers reported on the Food Distribution Program on Indian Reservations (FDPIR), which provided a monthly package of both perishable and non-perishable foods, and nutrition education, to low-income Native American households (*n* = 1053) [47, 48]. Food security survey and discussion group data showed the FDPIR was the only source of food for 38% of participants and that the food packages did not meet community needs; 34% of households initially reported low food security and 22% continued to have very low food security throughout the program [47]. Mucioki and colleagues’ case review using interviews and focus groups found only 151 households received the FDPIR, with 275 accessing other food assistance, and 242 not receiving any food assistance; the packages were seen as useful, but failed to alleviate food insecurity [48]. Evaluations found participants desired more fresh fruit, vegetables and traditional foods [48], and highlighted low eligibility and distribution issues [47, 48].

A program providing breakfast, and nutrition education, in two schools with high Aboriginal student populations in Western Australia was described [49]. Qualitative evaluation found that the food was provided passively to children, was not necessarily nutritious, and there was little evidence of health education. The authors recommended that schools should explore arrangements with local growers/shop owners to secure support for the program [49].

An included paper described a pilot study of “Eat Fresh”, a program delivering weekly boxes of recommended servings of fresh fruits and vegetables, along with nutrition education, to low-income Native American adults in the Montana-Flathead Reservation over 6 weeks [45]. This study assessed change in dietary habits and health perception, and in reported dietary intake and biomarkers. There was a reported improvement in variety of intake of fruit and vegetables and overall diet quality; however, over the intervention period, BMI and blood pressure increased. The authors noted the need for multi-strategy, holistic dietary interventions and a focus on the whole diet rather than just fruit and vegetables, given the lack of economic access to other foods and lack of time, resources and ‘hardware’ for food preparation and cooking. Results confirmed the need for multiple impact and outcome indicators to be assessed in evaluation. The authors recommended collaboration with the First Nations Community Advisory Board for co-design of subsequent intervention and feedback [45].

### Healthy food vouchers provided to community members/priority groups

Four papers evaluated programs providing food vouchers, mainly just for fruit and vegetables, to community members, rather than food; although one, as noted above, provided a $15 voucher for fruit and vegetables for each eligible child together with a monthly food box [46].

Fruit and vegetable prescriptions (FVRx) were provided to families of 243 Navajo children [50], with vouchers redeemable for fruit, vegetables, and traditional foods from participating retailers. Values were low: US$1 per household member per day, with a maximum value of $5 per day. Nearly two-thirds (65%) of participating children were retained in the program for more than 6 months. Information collected included reported food security (which increased from 18 to 35%), fruit and vegetable consumption, and child height and weight. While the results were promising, the authors noted several confounders, and concluded it was not possible to isolate or attribute outcomes [50].

Also in the USA, vouchers for fruit and vegetables were added to the food packages distributed by the Women Infant Children (WIC) program to the small cash value of US$6 for children and $10 for pregnant, breastfeeding, and postpartum women per month. Process data only were collected, with no significant outcomes noted in the Native American population [51]. The authors suggested several barriers contributed to the low redemption rate, and noted the need for further research.

In Australia, $10 vouchers for fruit and vegetables were provided to Indigenous women and children in several remote communities in Northern Australia over 32 weeks in two phases of different minimum spends [52]. Qualitative interviews and store sales data showed reduced sales of fruit (7%) and vegetables, and overall food and drinks, but it is not clear how population numbers were accounted. The median voucher redemption rate was 29% and was highest (44%) in the week when the project staff promoted the program with cooking demonstrations in store. Lack of support from retail store staff was seen as a barrier. The authors suggested loyalty cards may be more effective than paper vouchers, as might inclusion of a greater variety of healthy foods and increased program promotion.

### Increased income to community members for food purchases

To provide support over the holiday break to children who received school-based nutrition programs, the “Summer Electronic Benefit Transfers for Children” (SEBTC) program provided a cash benefit of US$60/child/month in 42,000 households in 14 sites in two Tribal Nations in the USA [53]. Data collected via food security and food frequency questionnaires showed the rate of very low food security was one-third lower in households receiving SEBTC, and that children in households receiving SEBTC consumed more healthy foods, including fruit and vegetables. Impacts in WIC sites where purchases were restricted to healthy foods were at least twice as large as those in Supplemental Nutrition Assistance Program (SNAP) sites where any foods or drinks could be purchased with the benefits.

### Government strategies and policies (not otherwise described above)

Five included publications reported evaluations of four different government strategies and policies as detailed below: a small tax waiver on healthy foods in the USA [54]; a National Food Security Strategy targeted to First Nations communities in Australia [55]; a compulsory income management program under the “Northern Territory Intervention” in Australia [56, 58]; and a community economic development program in Canada [57].

The Navajo Nation Healthy Diné Nation Act introduced in 2014 combined a 2% tax on foods of “minimal-to-no-nutritional value” with a waiver of 5% sales tax on healthy foods (including water, fresh fruits and vegetables and nuts) [54]. The hypothecated tax revenue was directed to local community wellness projects. Impacts were assessed by surveys of 51 Navajo stores and 20 stores in border towns, which collected data on pricing, food availability and in-store promotion. Over 6 years, after adjusting for inflation, the average cost per item of fresh fruit decreased by 13% in Navajo stores and increased in border stores, resulting in comparable prices in the stores in 2019. However, pricing trends among vegetables and other healthy foods were inconsistent.

A performance audit of Australia’s National Food Security Strategy for remote Indigenous communities, which included strategies to improve affordability of healthy foods in communities, found the initial trials were incomplete and no evidence that planned initiatives had been implemented at scale in any remote retail stores [55].

Two papers evaluated relevant aspects of the Northern Territory (NT) New Income Management policy, which quarantined 50% of income support and family assistance payments, and 100% of any lump sum payments, to Aboriginal people living in remote areas of the NT via an EFTPOS “BasicsCard” that could be used only for items considered essential by the government, such as food and clothes. Analysis of stores sales data from 10 community stores over 3 years (October 2006 to September 2009) found no effect on fruit and vegetable turnover [58]. Another evaluation identified the only change in spending patterns was a slight improvement in the reported incidence of running out of money for food for those on Voluntary Income Management; this was not seen for those on the compulsory program [56].

A community economic development program in Canada that promoted management of, and increased access to, traditional food systems was found to improve food security, assessed by changes in food price data and household food security surveys in 14 communities [57]. Community empowerment and control was noted as a key success factor [57].

## Promising interventions

Review of available impact and outcome evaluations identified six promising initiatives (Table 4). These included providing a box of shelf stable foods and voucher for fresh fruit and vegetables monthly [46] and prescription of vouchers for fruit, vegetables and traditional foods (“FVRx”) [50]. A third, the “Thrive” program, offered and promoted healthy meals and snacks in community stores at below the cost of unhealthy alternatives [25, 26]. Increasing income available for food via funds directly into community member’s bank accounts at times when school nutrition programs were not available [53], and selling discounted healthy foods from a mobile van visiting remote communities [34] also appeared to have merit. Finally, country food programs increasing access to traditional foods improved food security in some communities in Canada [57]. If barriers could be addressed, other programs that could be considered included “SHOP@RIC”, which tested discounting prices of selected healthy choices in remote community stores by 20% [27, 29, 30].

Evaluations of the most effective programs identified similar enablers of success (as noted in Table 4), including community co-design, control and empowerment; optimal promotion of the program throughout communities; and inclusion of a wide range of healthy foods (rather than only vegetables and fruit) and including traditional foods where possible.

Common barriers also were identified, including inadequate duration of the study; inadequate level of subsidisation or “dose” of intervention; lack of economic access to foods other than fruit and vegetables (when only fruit and vegetables were provided); lack of access to the resources and infrastructure required for cooking, food preparation and storage; inadequate promotion of the project within community; insufficient community consultation; and, particularly, imposition of the program from ‘above’ (Table 4). Several studies noted the complexity of the food supply system, which made it difficult to measure and assess the impact of confounding factors in study outcomes, including the impact of ‘education’ programs, even when these were randomised as a feature of the study design [33, 35, 46, 50].

## Discussion

### The context and heterogeneity of economic interventions

The number and diversity of approaches to improve economic access to healthy diets in First Nations communities in high income colonised countries identified in this systematic scoping review reflects the long-term and widespread nature of this problem, which has exacerbated during the current global cost-of-living crisis [59]. The results of the review were heterogenous both in terms of the type of intervention and the process, impact and outcome metrics qualitatively and quantitatively described. While most studies targeted household-level economic access to healthy foods, some studies, such as those applying subsidies throughout the food supply chain, were more focussed on community-level food security. Given the variation, there was a need for the broader context to be well described to aid assessment of both significance of any results and the relevance of these to other settings. For example, the community economic development program facilitating traditional food programs [57] would be unlikely transferable to all communities. Also, few Australian-based studies noted the universal policy of exemption of “basic, healthy foods” from 10% GST for all consumers, nationally. This, and the inclusion of alcohol and takeaway foods in assessment of cost of habitual diets, can make these more expensive than healthy diets in Australia [60], which should be considered when interpreting results of interventions to improve economic access to healthy foods.

### Study design

Study design varied from opportunistic ‘real world’ evaluations [19, 31, 56] to well-designed RCTs [25, 26, 29]. Major parameters that differed between studies and have been noted previously included duration [61]. Given the long consultation and ‘lead-time’ in many nutrition studies in First Nations’ communities, the need to fully promote strategies and activities, the entrenched inter-generational disadvantage, and seasonal variation of dietary intake, it could be expected that duration of 12 months or more would be required to achieve measurable impact [62]. Another key variable was the level, magnitude or ‘dose’ of the monetary value of the food, voucher or subsidy [63, 64]. For example, the hypothecated tax of 2% applied to unhealthy foods in Navajo communities [54], and the additional US$6 per child per month to recipients of WIC assistance [51], were very small compared to the level of at least 20% taxation on sugary drinks that the World Health Organization recommends for success [65].

Provision of free healthy food and/or vouchers for healthy food to community members was one of the most common, but also the most diverse, approaches (eight papers/reports describing seven studies). These tended to be smaller studies of relatively short duration and were most frequently implemented from the ‘bottom up’, with most benefitting from co-design of interventions with the communities involved. Provision of price discounts on healthy foods made available to community members via several channels, particularly via community retail stores, was also described frequently (*n* = 13 papers/reports describing seven studies). These studies tended to employ strong research design, were larger and ran for longer periods than those providing free healthy food and/or vouchers, but also had good levels of community involvement and support.

### Common challenges

Conversely, although eight papers/reports described subsidies paid directly to retail stores, suppliers and/or producers, these focussed on just two interventions in Canada, one of these targeting transport specifically. Neither was developed in co-design with communities, there was low population awareness of the programs, and, where assessed, food security worsened, as the subsidies frequently did not flow through the food chain to consumers. These results highlight specifically the challenges around the commercial determinants of health [66], illustrating that direct subsidies to food industry groups are likely to benefit industry shareholders, but unlikely to benefit vulnerable consumers [37, 39, 43].

In several evaluated programs offerings were restricted to vegetables and fruit only; for example, 80% of vouchers provided to community members were redeemable just for fruit and vegetables. Self-reported intake, or biomedical indicators, of fruit and vegetable consumption increased in some studies, but did not occur together in any study, highlighting the need for multiple evaluation measures at impact and outcome level. Several studies described barriers such as lack of resources to access other foods to combine with the vegetables, and lack of cooking facilities, infrastructure and ‘health hardware’ [67].

Several interventions involved nutrition education in at least one arm of the study, as well as economic interventions (Table 4). None of these found any positive effect of nutrition education on dietary change. This confirms consistent findings that lack of resources and infrastructure, rather than any lack of nutrition knowledge, is the major contributor to low economic access to healthy foods in First Nations’ communities [9, 10, 68].

Of the included studies, the two providing subsidies along the food supply chain [37–44] were least effective. Both these were led by governments with industry partners, and the multiple evaluations showed little community support and highlighted risks with the subsidies not being passed onto consumers [37, 39, 43].

There is strong evidence of the need for a strengths-based approach to tackle food security in remote First Nations communities that builds on lived experience and Indigenous ways of knowing, doing and being [9]. Programs that undermine community strengths, such as those imposing a colonising view of nutrition and food-ways, are unlikely to be successful [62].

### Most promising strategies relevant to the APY lands

The co-design workshop with members of the NPYWC Anangu research team and service providers held in Alice Springs in Central Australia in November 2022 provided the opportunity to privilege First Nations’ perspectives while collectively considering the findings of the literature review. Recently, our team’s Indigenist research methodologies, including ‘yarning’, have been commended in a relevant scoping review [69]. A copy of the presentation of the findings is included at Supplementary File 3. Review of the included studies highlighted that different country and community contexts were essential to consider in identification of the most promising interventions for testing elsewhere, including on the APY Lands. Workshop participants discussed the promising strategies and impacts and agreed that the following five interventions would be discussed further with other community leaders and members, before potential trial on the APY Lands.

Of most interest was the “Thrive” project as it was the only intervention in the category of price discount on healthy foods via retail stores that included healthy ready-to-eat meals and snacks. Participants felt that the availability of single-serve healthy meals would help overcome limitations of inadequate housing, cooking facilities, and some challenges around social obligations experienced in their communities [9]. The use of mobile vans [34] was also considered promising, particularly where store management groups were “not listening” to community members or not fully implementing agreed nutrition policies in stores [9]. There was also support for regular supply of a free box of healthy foods and/or vouchers for fresh produce [46], consistent with previous unpublished recommendations to Nganampa Health from the National Center for Social and Economic Modelling. However, as with clinical prescriptions for fruit, vegetables and traditional foods [50], which was also supported, lack of functional cooking and storage facilities and other “health hardware” [9, 67] was identified as a potential barrier. Participants also noted that, given the reduced availability of traditional foods throughout the APY Lands due to incursion by buffel grass (*Cenchrus ciliaris*), feral animals and changing fire regimes [9, 10], that prescriptions for traditional foods would have, unfortunately, limited impact on the APY Lands. Direct cash transfer [53] was also a welcomed idea, and prompted discussion about evaluation of the natural experiment of increased welfare benefits in the early days of the COVID 19 pandemic.

Workshop participants noted that the literature review helped identify a broad range of possible approaches to improve affordability of healthy foods on the APY Lands and highlighted barriers and enablers of effective strategies to improve economic access to healthy food in comparable communities. Agreed next steps included facilitating wider consideration of and consultation on the shortlist of approaches to test, and in which communities on the APY Lands, with community leaders and members, Nganampa Health Council and other service providers. The critical role of members of the NPYWC Anangu research team in leading this broader community consultation and development of recommendations was supported by all participants.

### Study limitations

The study was limited to four nations with similar histories and political systems; it is possible that additional interventions in other First Nations communities may exist. Also, given the current global cost-of-living crisis [59], there is a need for urgent action to improve economic access to healthy foods in First Nations communities, so several relevant papers are likely to have been published after May 2022 and further studies are likely to be underway. For example, these include a co-designed healthy food price discount trial using EFTPOS ‘smart cards’ in Central and Northern Australia [70]. Therefore, before acting on the results of the current review, an additional, targeted search of papers could be warranted.

## Conclusions

A variety of possible approaches to increase affordability of healthy foods in remote First Nations communities in high income countries was identified through the systematic scoping review. Of the 21 interventions identified, six were deemed promising, and of those five reflected the voices and experiences of Anangu [9, 68], and were considered relevant for further consideration by and consultation with community leaders, members and service providers on the APY Lands. All authors and workshop participants also agreed that further co-design workshops should be held in the communities on the APY Lands to identify the most relevant, promising and popular approach for potential testing to increase economic access to healthy foods in communities. The findings highlight potential approaches to improve economic access to healthy foods in other First Nations communities in high-income colonised countries too.

### Supplementary Information

**Additional file 1.** Detailed search strategies and results (DOCX 19 kb).

**Additional file 2.** Data extraction spreadsheets (XLSX 52 kb).

**Additional file 3.** Copy of slides presenting findings of literature review to NPYWC Anangu research team and service providers (co-design workshop), Alice Springs, November 2022 (PDF 1939 kb).

## Data Availability

All data generated or analysed during this study are included in this published article and its supplementary information files.

## Footnotes

1 The year food security was redefined at the World Food Summit to include ‘physical and economic access to sufficient, safe and nutritious food’ (Rome Declaration on World Food Security).
