# Supplementary material for: Improving economic access to healthy diets in first nations communities in high-income, colonised countries: a systematic scoping review
Source: Nutr J. 2024 Jan 16;23:10. doi: 10.1186/s12937-023-00895-0 (PMC10790425; doi:10.1186/s12937-023-00895-0)
Supplement: Supplementary file 1 — Additional file 1. Detailed search strategies and results (DOCX 19 kb). [file 12937_2023_895_MOESM1_ESM.docx]

**Supplementary File 1: Detailed search strategies and results**

**Results of searches of academic databases**

| **Database** | **Provider** | **Search date** | **Results (n)** |
| --- | --- | --- | --- |
| PubMed | US National Library of Medicine | 20 May 2022 | 323 |
| Web of Science | Clarivate | 22 May 2022 | 210 |
| Cochrane | Cochrane Library | 20 May 2022 | 97 |
| EconLit | EBSCOhost Research Databases | 20 May 2022 | 18 |
| Social Science | ProQuest | 20 May 2022 | 8 |
| Indigenous collection | Informit | 20 May 2022 | 17 |
| Australian Public Affairs (APAFT) | Informit | 20 May 2022 | 19 |
| ATSIHEALTH | Informit | 20 May 2022 | 1 |
| **Total** |  |  | 693 |

**Results of searches of selected websites including research hubs**

| **Website** | **Results screened** | **Full records assessed for eligibility** | **Included** |
| --- | --- | --- | --- |
| Australian Institute of Aboriginal and Torres Strait Islander Studies | 104 | 0 | 0 |
| Australian Indigenous Health InfoNet | 43 | 10 | 1 |
| Australian Government Department of Health | first 50 | 1 | 0 |
| National Indigenous Australians Agency | 6 | 0 | 0 |
| Indigenous.gov.au | 21 | 0 | 0 |
| Australian Institute of Family Studies | 23 | 3 | 1 |
| Centre for Aboriginal Economic Policy Research | 16 | 8 | 0 |
| Indigenous studies portal research tool (iPortal) (Canada) | 69 | 11 | 1 |
| National Collaborating Centre for Indigenous Health (Canada) | 4 | 2 | 0 |
| Government of Canada | first 50 | 12 | 1 |
| Indigenous Services Canada | first 50 | 1 | 0 |
| Nutrition North Canada |  | 5 | 1 |
| PROOF (Food Insecurity Policy Research program) (Canada) | 75 | 5 | 0 |
| Food Secure Canada | 70 | 7 | 2 |
| US Economic Research Service (US Department of Agriculture) | first 50 x 5 | 10 | 0 |
| US Food and Nutrition Service (US Department of Agriculture) | # | 1 | 0 |
| US First Nations Development Institute | first 50 | 2 | 0 |
| US National Institute of Food and Agriculture | 30 | 0 | 0 |
| NZ Ministry of Health – Maori Health | 96 | 0 | 0 |
| NZ Ministry of Health | 77 | 0 | 0 |
| The Hub (repository for NZ Government social science research) | 38 | 0 | 0 |
| Google | first 50 x 5 | 4 | 1 |
| **TOTALS** | **1,372** | **83** | **9** |

*# Unable to access linked pages from this website (region locked); Google searches were conducted for document titles in results that appeared potentially relevant*

**Example of full search strategy: PubMed**

1 food security[MeSH Terms] 405

2 "food secur*" OR "food insecur*" OR "food sufficien*" OR "food insufficien*" OR "food access*" OR "food afford*" OR "food sovereign*" OR "food pric*" 20,646

3 (diet OR fruit OR vegetable OR grocer* OR nutrition* OR meal) AND (afford* OR pric* OR access*) 44,043

4 intervention OR policy OR policies OR strateg* OR evaluat* 12,477,020

5 income OR "cost of living" OR poverty OR financ* OR budget* OR payment OR benefit OR money OR cash OR supplement* OR voucher OR coupon OR expen* OR spend* OR purchas* OR buy OR subsid* OR welfare OR "social security" OR "social support" OR "social protection" OR "social enterprise" OR tax OR taxation 2,494,029

6 Aborigin* OR Torres Strait Island* OR Indigen* OR "First Nation*" OR Maori OR Inuit OR Metis OR "Native Canadian" OR "Native American" OR "American Indian" OR "Alaska Native" OR "first people*" OR "native group*" 103,591

7 Australia* OR "New Zealand" OR "NZ" OR Canada OR "United States" OR "US" OR "USA" OR "North America" 9,130,309

8 ((food security[MeSH Terms]) OR ("food secur*" OR "food insecur*" OR "food sufficien*" OR "food insufficien*" OR "food access*" OR "food afford*" OR "food sovereign*" OR "food pric*")) OR ((diet OR fruit OR vegetable OR grocer* OR nutrition* OR meal) AND (afford* OR pric* OR access*)) 61,688

9 ((((((food security[MeSH Terms]) OR ("food secur*" OR "food insecur*" OR "food sufficien*" OR "food insufficien*" OR "food access*" OR "food afford*" OR "food sovereign*" OR "food pric*")) OR ((diet OR fruit OR vegetable OR grocer* OR nutrition* OR meal) AND (afford* OR pric* OR access*))) AND (intervention OR policy OR policies OR strateg* OR evaluat*)) AND (income OR "cost of living" OR poverty OR financ* OR budget* OR payment OR benefit OR money OR cash OR supplement* OR voucher OR coupon OR expen* OR spend* OR purchas* OR buy OR subsid* OR welfare OR "social security" OR "social support" OR "social protection" OR "social enterprise" OR tax OR taxation)) AND (Aborigin* OR Torres Strait Island* OR Indigen* OR "First Nation*" OR Maori OR Inuit OR Metis OR "Native Canadian" OR "Native American" OR "American Indian" OR "Alaska Native" OR "first people*" OR "native group*")) AND (Australia* OR "New Zealand" OR "NZ" OR Canada OR "United States" OR "US" OR "USA" OR "North America") 337

10 ((((((((food security[MeSH Terms]) OR ("food secur*" OR "food insecur*" OR "food sufficien*" OR "food insufficien*" OR "food access*" OR "food afford*" OR "food sovereign*" OR "food pric*")) OR ((diet OR fruit OR vegetable OR grocer* OR nutrition* OR meal) AND (afford* OR pric* OR access*))) AND (intervention OR policy OR policies OR strateg* OR evaluat*)) AND (income OR "cost of living" OR poverty OR financ* OR budget* OR payment OR benefit OR money OR cash OR supplement* OR voucher OR coupon OR expen* OR spend* OR purchas* OR buy OR subsid* OR welfare OR "social security" OR "social support" OR "social protection" OR "social enterprise" OR tax OR taxation)) AND (Aborigin* OR Torres Strait Island* OR Indigen* OR "First Nation*" OR Maori OR Inuit OR Metis OR "Native Canadian" OR "Native American" OR "American Indian" OR "Alaska Native" OR "first people*" OR "native group*")) AND (Australia* OR "New Zealand" OR "NZ" OR Canada OR "United States" OR "US" OR "USA" OR "North America")) NOT (animal[Filter])) AND (("1996/01/01"[Date - Publication] : "3000"[Date - Publication])) 318
