# Supplementary material for: Improving economic access to healthy diets in first nations communities in high-income, colonised countries: a systematic scoping review
Source: Nutr J. 2024 Jan 16;23:10. doi: 10.1186/s12937-023-00895-0 (PMC10790425; doi:10.1186/s12937-023-00895-0)
Supplement: Supplementary file 3 — Additional file 3. Copy of slides presenting findings of literature review to NPYWC Anangu research team and service providers (co-design workshop), Alice Springs, November 2022 (PDF 1939 kb). [file 12937_2023_895_MOESM3_ESM.pdf]

Researcherenye Wappayalawangka
Central Australia Academic Health Science Network

Ngaanyatjarra Pitjantjatjara Yankunytjatjara Women's Council

Nganampa Health Council

The University of Queensland Australia
Create Change

Improving economic access to healthy diets in Aboriginal and Torres Strait Islander communities in Australia:

A review of research in remote First Nations communities in Australia and other countries

Medical Research Future Fund

What we already knew

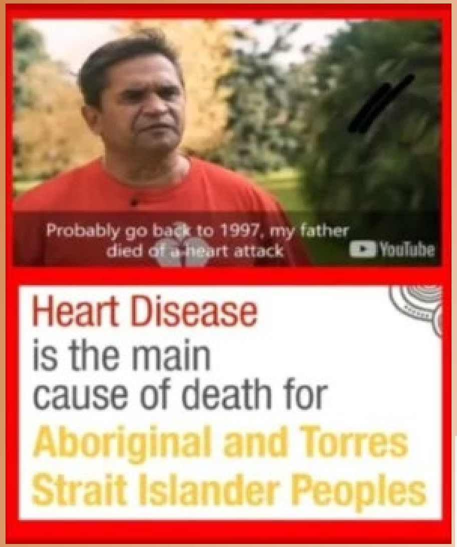

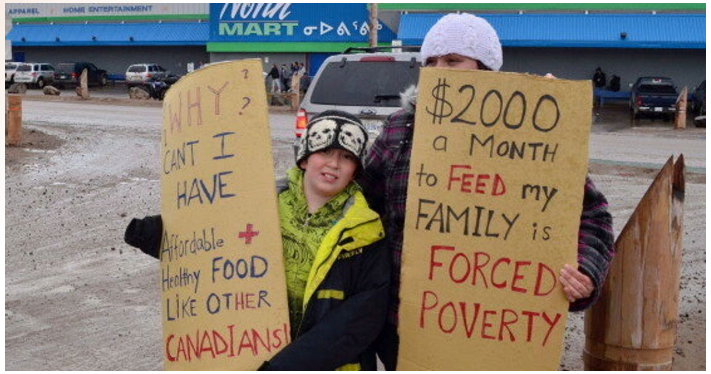

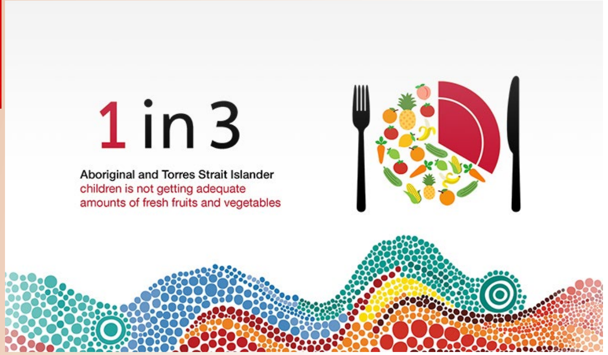

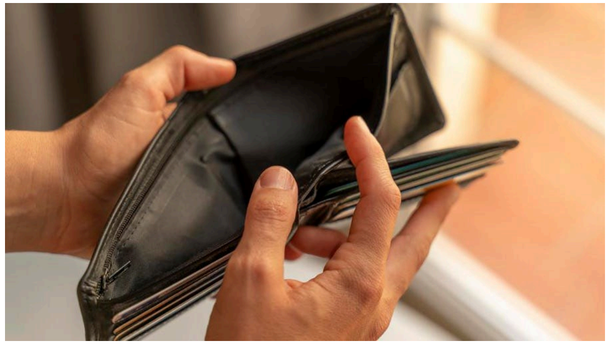

Image credits: NACCHOcommunique.com; RACGP; Huffington Post (CP/Aaron Watson); stock image

Ngaanyatjarra Pitjantjatjara Yankunytjatjara Women's Council
NGANAMPA HEALTH COUNCIL
THE UNIVERSITY OF QUEENSLAND AUSTRALIA
CREATE CHANGE

What we wanted to find out

- What could help people to buy more healthy food and drinks?
  - Government policy/system change

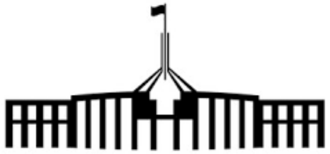

  - Discounts on healthy food and drinks

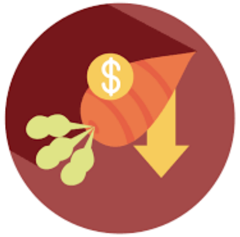

  - More income or free healthy food

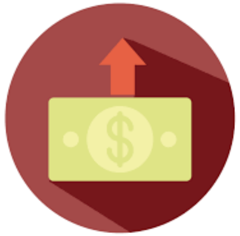

Ngaanyatjarra Pitjantjatjara Yankunytjatjara Women's Council

NGANAMPA HEALTH COUNCIL

The University of Queensland Australia

Create Change

What we did

Ngaanyatjarra Pitjantjatjara Yankunytjatjara Women's Council

Researcherenye Wappayalawangka
Central Australia Academic Health Science Network

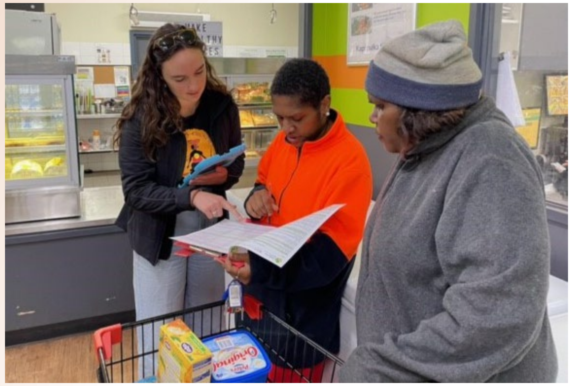

NGANAMPA HEALTH COUNCIL

THE UNIVERSITY OF QUEENSLAND
AUSTRALIA
CREATE CHANGE

What we did

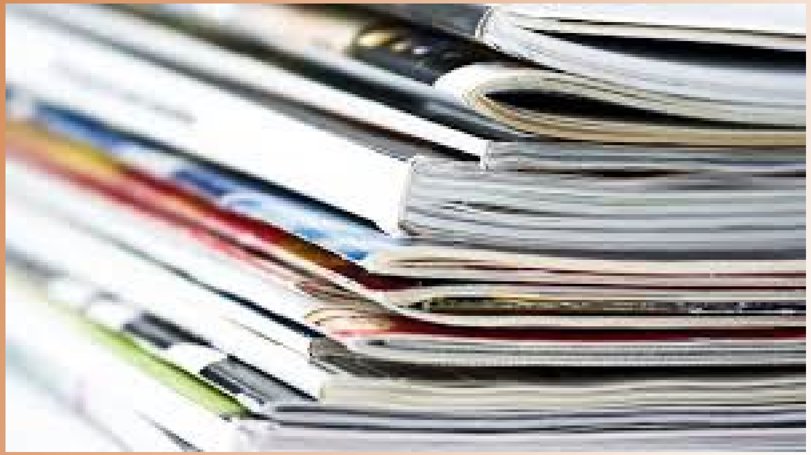

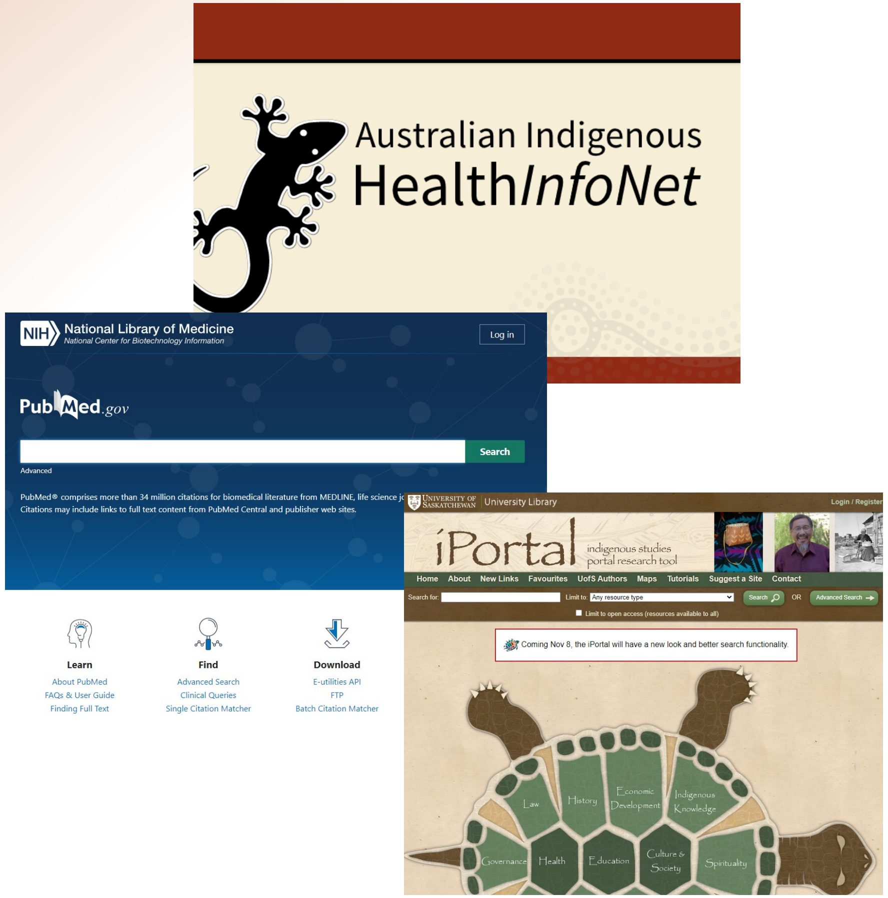

1,968 records → 212 possible → 35 reports

Ngaanyatjarra Pitjantjatjara Yankunytjatjara Women's Council

NGANAMPA HEALTH COUNCIL

The University of Queensland Australia
Create Change

What we found

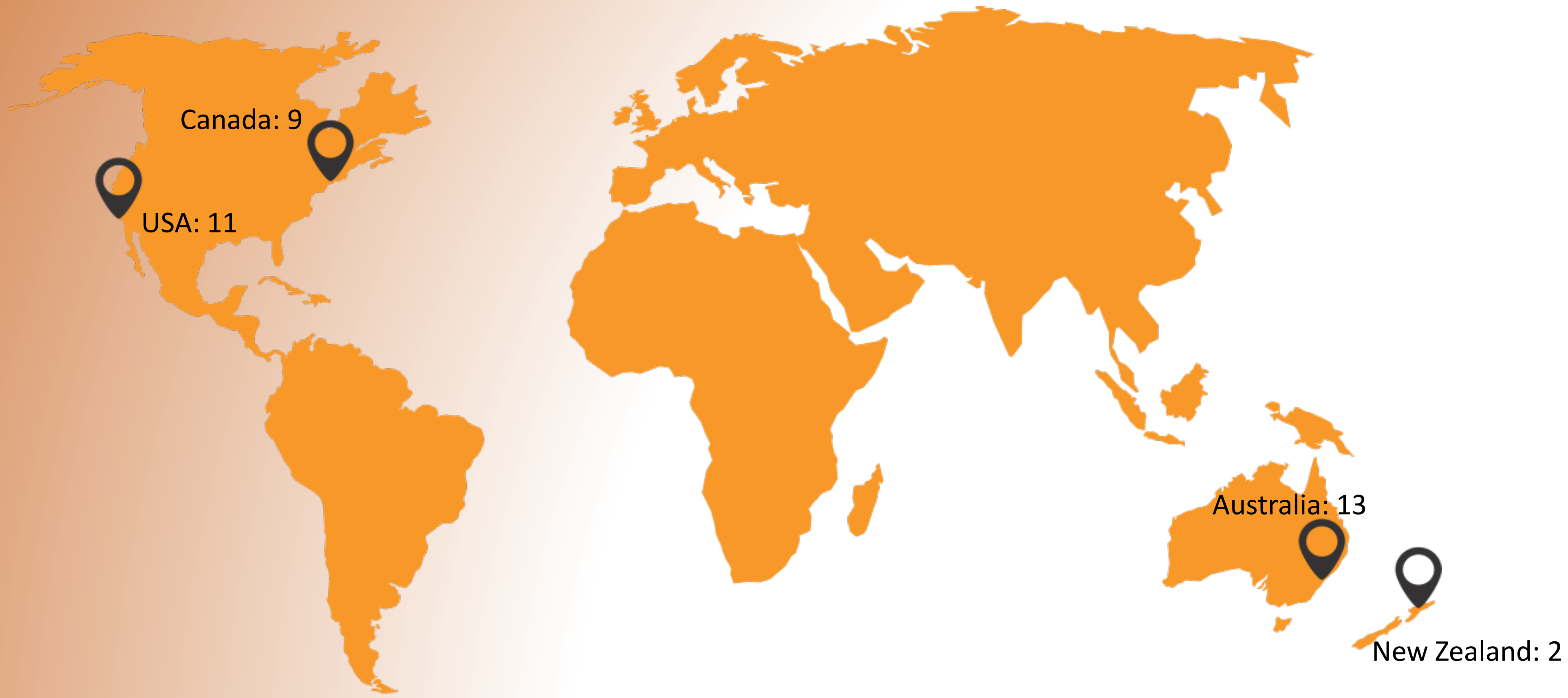

Ngaanyatjarra Pitjantjatjara Yankunytjatjara Women's Council

NGANAMPA HEALTH COUNCIL

THE UNIVERSITY OF QUEENSLAND AUSTRALIA
CREATE CHANGE

What we found

- Government strategy, policy (tax), or subsidies

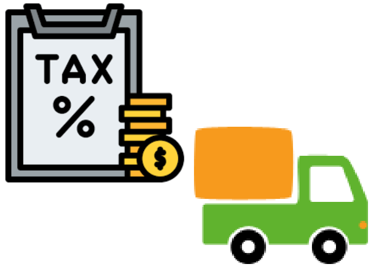

- Healthy food discounts in stores

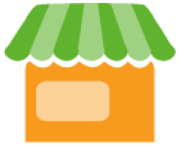

- Discounted healthy food in communities

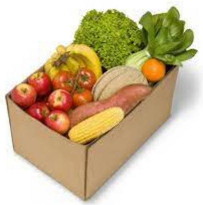

- More income or
  free healthy food or food vouchers

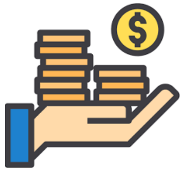

Ideas tried in other places

- Tax on junk food and no tax on healthy food

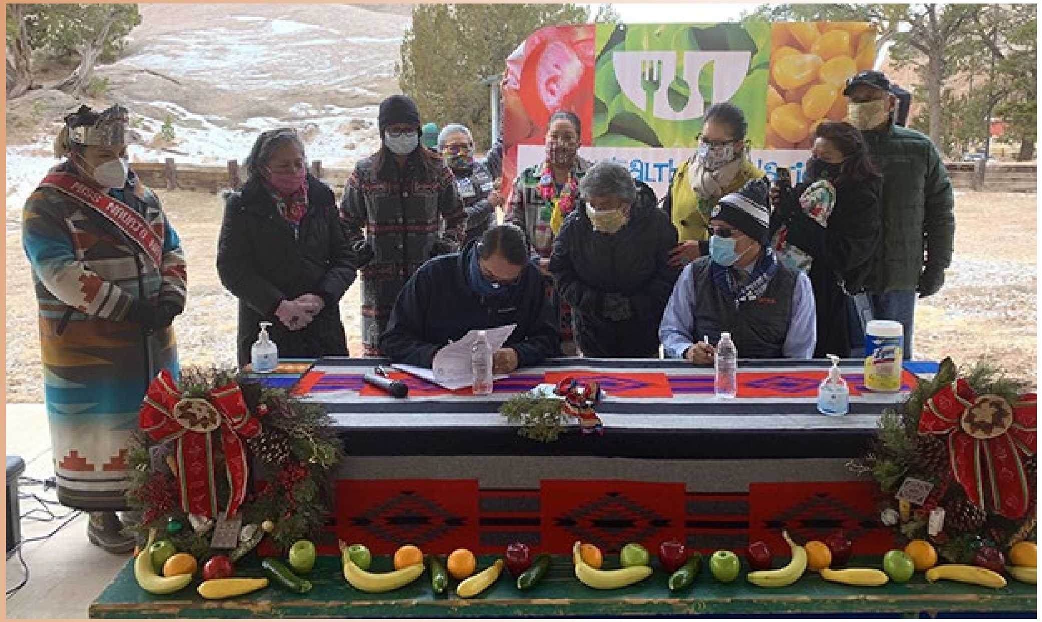

Image credit: Navajo Nation Council

In Australia:

☑ No GST on healthy foods and drinks

☒ Extra tax on junk food and/or sugary drinks

Ngaanyatjarra Pitjantjatjara Yankunytjatjara Women's Council

NGANAMPA HEALTH COUNCIL

THE UNIVERSITY OF QUEENSLAND AUSTRALIA

CREATE CHANGE

Ideas tried in other places

- Subsidies to stores

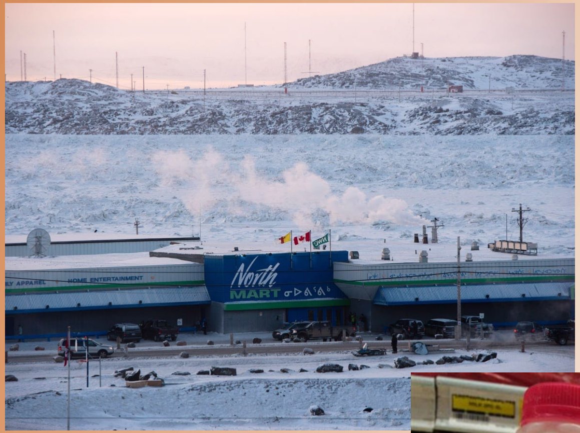

Northern region of Canada

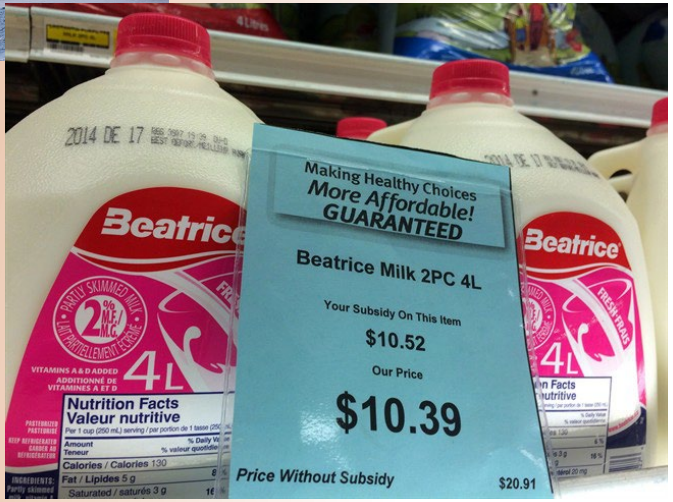

Subsidies to remote stores in Australia:

☑ Outback Stores

? ALPA

Is this happening on the APY Lands?

Would it work?

Ngaanyatjarra Pitjantjatjara Yankunytjatjara Women's Council

NGANAMPA HEALTH COUNCIL

The University of Queensland
Australia
CREATE CHANGE

Ideas tried in other places

- Discounted healthy food and drinks in stores

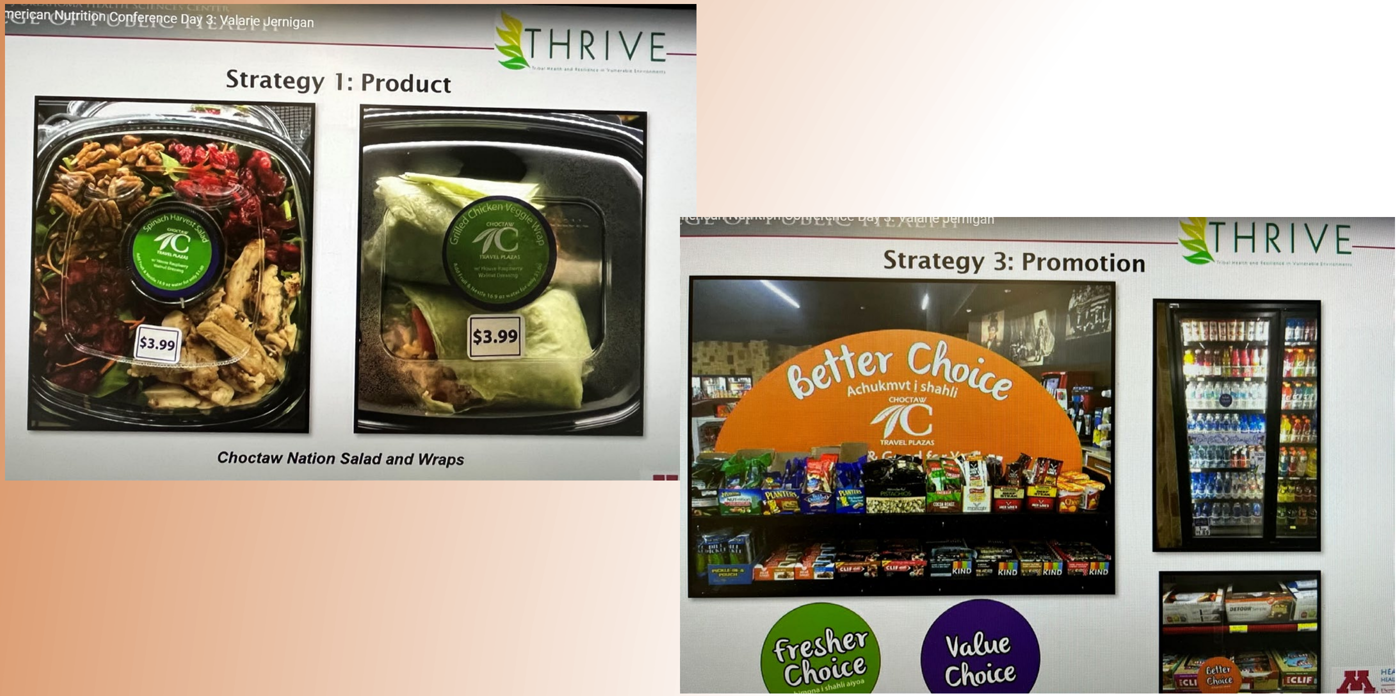

Is this happening on the APY Lands?

Would it work?

Image credit: Slides from presentation by THRIVE study lead researcher Dr Valarie Blue Bird Jernigan, available at: https://indigenoushealth.com/knowledge-center/media/

Ngaanyatjarra Pitjantjatjara Yankunytjatjara Women's Council
NGANAMPA HEALTH COUNCIL
THE UNIVERSITY OF QUEENSLAND AUSTRALIA
CREATE CHANGE

Ideas tried in other places

- Bring discounted healthy food and drinks to communities, other than through stores

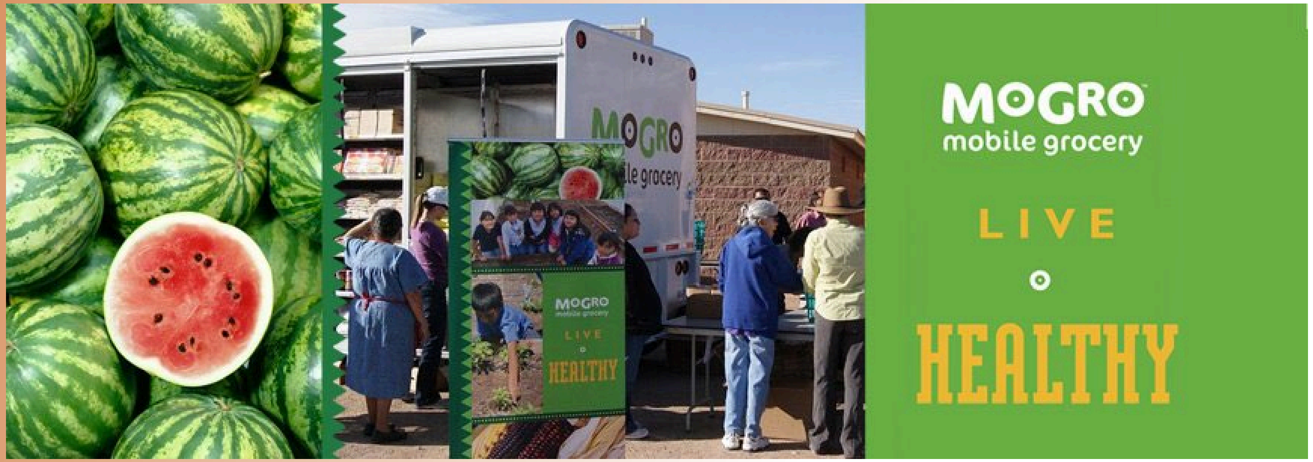

Is this happening on the APY Lands?

Would it work?

Image credit: MoGro Facebook page

Ideas tried in other places

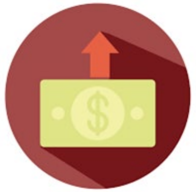

- Free healthy food or vouchers (spending less on food)

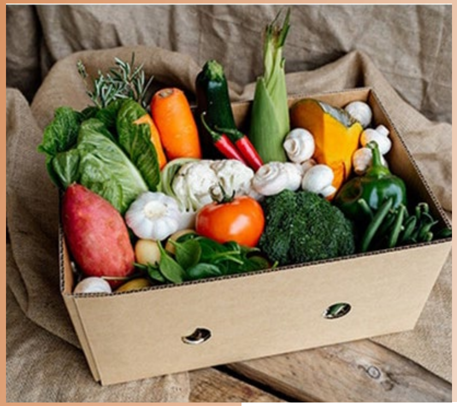

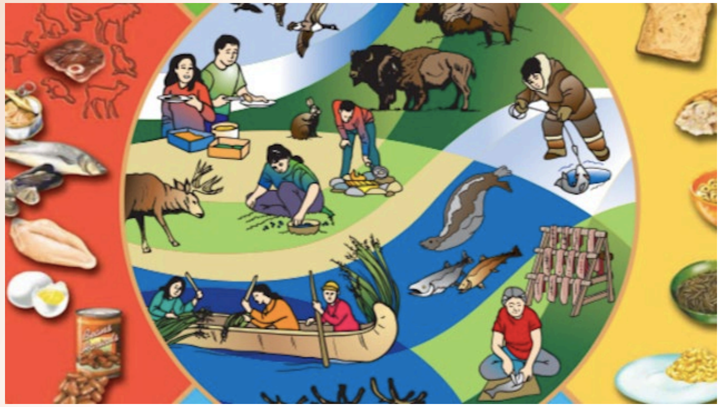

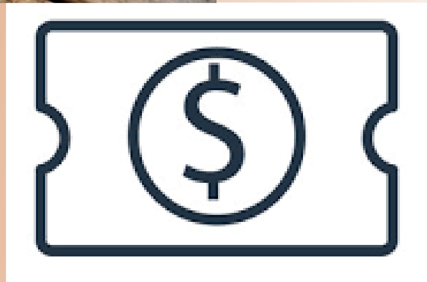

Is this happening on the APY Lands?

Would it work?

Ngaanyatjarra Pitjantjatjara Yankunytjatjara Women's Council

NGANAMPA HEALTH COUNCIL

THE UNIVERSITY OF QUEENSLAND AUSTRALIA

CREATE CHANGE

Ideas tried in other places

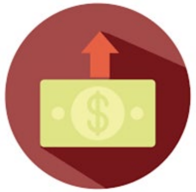

- ? More income

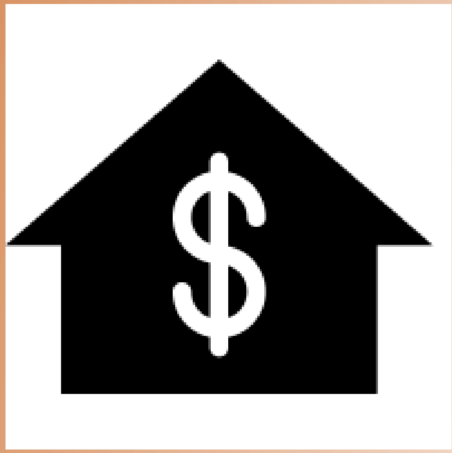

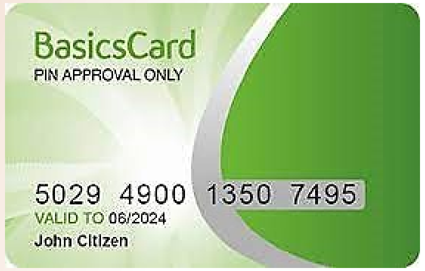

Is this happening on the APY Lands?

Would it work?

Ngaanyatjarra Pitjantjatjara Yankunytjatjara Women's Council

Nganampa Health Council

The University Of Queensland Australia
Create Change

What would help?

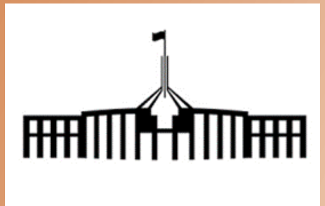

- Tax on unhealthy food/drinks?
- Subsidies to stores?
- Transport subsidies?

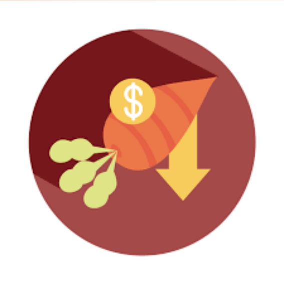

- Price discounts on healthy food/drinks?
- Discounted ready-to-eat meals and snacks?

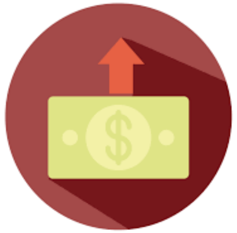

- More benefits?
- Free healthy food?
- Vouchers to get healthy food from store?

Ngaanyatjarra Pitjantjatjara Yankunytjatjara Women's Council

NGANAMPA HEALTH COUNCIL

THE UNIVERSITY OF QUEENSLAND AUSTRALIA

CREATE CHANGE
